# Ghrelin Pre-treatment Attenuates Local Oxidative Stress and End Organ Damage During Cardiopulmonary Bypass in Anesthetized Rats

**DOI:** 10.3389/fphys.2018.00196

**Published:** 2018-03-09

**Authors:** Vijayakumar Sukumaran, Hirotsugu Tsuchimochi, Yutaka Fujii, Hiroshi Hosoda, Kenji Kangawa, Tsuyoshi Akiyama, Mikiyasu Shirai, Eisuke Tatsumi, James T. Pearson

**Affiliations:** ^1^Department of Artificial Organs, National Cerebral and Cardiovascular Centre Research Institute, Suita, Japan; ^2^Cardiac Physiology, National Cerebral and Cardiovascular Centre Research Institute, Suita, Japan; ^3^Department of Clinical Engineering and Medical Technology, Niigata University of Health and Welfare, Niigata, Japan; ^4^Regenerative Medicine and Tissue Engineering, National Cerebral and Cardiovascular Centre Research Institute, Suita, Japan; ^5^Biochemistry, National Cerebral and Cardiovascular Centre Research Institute, Suita, Japan; ^6^Advanced Medical Research for Pulmonary Hypertension, National Cerebral and Cardiovascular Centre Research Institute, Suita, Japan; ^7^Department of Physiology, Monash Biomedicine Discovery Institute, Monash University, Clayton, VIC, Australia

**Keywords:** ghrelin, inflammation, organ damage, oxidative stress, cardiopulmonary bypass

## Abstract

Cardiopulmonary bypass (CPB) induced systemic inflammation significantly contributes to the development of postoperative complications, including respiratory failure, myocardial, renal and neurological dysfunction and ultimately can lead to failure of multiple organs. Ghrelin is a small endogenous peptide with wide ranging physiological effects on metabolism and cardiovascular regulation. Herein, we investigated the protective effects of ghrelin against CPB-induced inflammatory reactions, oxidative stress and acute organ damage. Adult male Sprague Dawley rats randomly received vehicle (*n* = 5) or a bolus of ghrelin (150 μg/kg, sc, *n* = 5) and were subjected to CPB for 4 h (protocol 1). In separate rats, ghrelin pre-treatment (protocol 2) was compared to two doses of ghrelin (protocol 3) before and after CPB for 2 h followed by recovery for 2 h. Blood samples were taken prior to CPB, and following CPB at 2 h and 4 h. Organ nitrosative stress (3-nitrotyrosine) was measured by Western blotting. CPB induced leukocytosis with increased plasma levels of tumor necrosis factor-α and interleukin-6 indicating a potent inflammatory response. Ghrelin treatment significantly reduced plasma organ damage markers (lactate dehydrogenase, aspartate aminotransferase, alanine aminotransferase) and protein levels of 3-nitrotyrosine, particularly in the brain, lung and liver, but only partly suppressed inflammatory cell invasion and did not reduce proinflammatory cytokine production. Ghrelin partially attenuated the CPB-induced elevation of epinephrine and to a lesser extent norepinephrine when compared to the CPB saline group, while dopamine levels were completely suppressed. Ghrelin treatment sustained plasma levels of reduced glutathione and decreased glutathione disulphide when compared to CPB saline rats. These results suggest that even though ghrelin only partially inhibited the large CPB induced increase in catecholamines and organ macrophage infiltration, it reduced oxidative stress and subsequent cell damage. Pre-treatment with ghrelin might provide an effective adjunct therapy for preventing widespread CPB induced organ injury.

## Introduction

**Figure 1:**
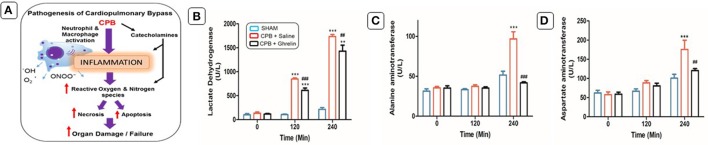
Schematic diagram showing the pathophysiological changes during the CPB **(A)**. Ghrelin treatment improved organ damage indices. Effects of ghrelin on LDH **(B)**, ALT **(C)**, and AST **(D)** before, and after 120 min and 240 min of CPB. The data shown are the mean ± SEM; *N* = 5 rats per group. Statistical differences were determined by two-way ANOVA followed by Tukey's test or Student's unpaired *t*-test. **p < 0.01, ***p < 0.001 vs. sham group; ^##^*p* < 0.01, ^###^*p* < 0.001 vs. CPB saline group.

Extracorporeal life support devices, such as cardiopulmonary bypass (CPB) preserve a patient's life by providing adequate oxygen supply and blood flow to vital organs. Despite the profound benefits that CPB has brought to patients around the world, it triggers a vigorous systemic inflammatory response that significantly increases the morbidity and mortality after CPB (Ng and Wan, 2012). This systemic inflammation is thought to be induced by contact of the blood components with the artificial surfaces of the CPB circuit (Hall et al., 1997). CPB induced inflammation contributes to the development of postoperative complications, including myocardial dysfunction, respiratory failure, renal and neurological dysfunction and can ultimately lead to the failure of multiple organs (Figure 1A; Gottesman et al., 2008; Heusch et al., 2012). In addition, plasma levels of catecholamines are increased during extracorporeal circulation in response to hypotension (Takewa et al., 1999) and crosstalk between immune cells and the sympathetic nervous system results in extracorporeal circulation induced sympathetic activation and potentiation of systemic inflammation (Pongratz and Straub, 2014).

Ghrelin bioactive peptide has a diverse range of physiological effects on neurohormonal control, metabolic homeostasis, and cardiovascular regulation (Nagaya and Kangawa, 2003; Schwenke et al., 2008; Soeki et al., 2008; Imazu et al., 2011; Callaghan et al., 2012). Recent evidence suggests that, ghrelin acts as a potent anti-inflammatory mediator both *in vitro* and *in vivo* and displays promising therapeutic benefits in the treatment of inflammatory diseases (Deboer et al., 2008; Erşahin et al., 2011). Ghrelin has been shown to have potential therapeutic benefit in patients with heart failure, as ghrelin administration significantly improved cardiac function and decreased systemic vascular resistance (Nagaya et al., 2001; Okumura et al., 2002; Nagaya and Kangawa, 2003), and improved left ventricular ejection fraction during exercise (Kishimoto et al., 2012). Ghrelin treatment reduced the inflammatory response, apoptosis and preserved cardiac pump function in a rat model of cardiopulmonary bypass through ghrelin receptor (GHSR1a) and Akt signaling (Cao et al., 2013). Further, several studies have shown that ghrelin greatly attenuates pathological increases in sympathetic nervous activity (Schwenke et al., 2008; Callaghan et al., 2012). Therefore, we hypothesized that ghrelin may have a potential benefit in preventing systemic inflammation and end organ damage induced by CPB. To test this hypothesis, we first investigated the benefit of bolus ghrelin administration during prolonged (4 h) CPB in the rat. Furthermore, we investigated the histological changes in the major organs. Next, we investigated the effects of a second bolus of ghrelin after a more clinically relevant period of CPB (2 h) on clinical markers of end organ damage to determine if maximal benefits are obtained by the single bolus of ghrelin.

## Materials and methods

### Animals

Animal protocols were approved by the Institutional Animal Care and Use Committee of National Cerebral and Cardiovascular Research Institute and conducted under the guidelines for the Care and Use of Laboratory Animals published by the US National Research Council. 16 weeks old Male Sprague-Dawley rats (450–480 g) were purchased from SLC, Japan. Animals were housed 3 per cage under controlled conditions (12:12 h-light dark cycles) and were fed *ad libitum* with water and standard food pellets (Oriental Yeast Co., Ltd, Tokyo, Japan). The rat CPB was applied via venoarterial bypass and performed following Fujii et al. (2015, 2016).

### Anesthesia, surgical preparation and CPB

The CPB model used in this study is considered to be equivalent to the established human CPB procedure, which is often associated with systemic inflammation and organ damage (Boyle et al., 1997; Fujii et al., 2015, 2016). We used a blood flow rate comparable to clinical reports, about 2.4 l/min/m^2^ of body surface area, with a cardiac index sufficient to adequately perfuse an adult patient during mild hypothermia and with 24% haematocrit levels (Alston et al., 2006; Santambrogio et al., 2009). In this study, the animals were anesthetized with isoflurane (2.0%) and placed in the supine position with a rectal thermocouple in place. Then, tracheal intubation was performed using a 14G cannula (Insyte BD Medical, Sandy, UT, USA) and rats were artificially ventilated (Model MA-01746, HARWARD APPARATUS, Holliston, USA). Ventilation was volume controlled at a frequency of 70/min, a tidal volume of 8–10 ml/kg body weight, and 40% inspired oxygen fraction. Rectal temperature was maintained at 36°C throughout the experiment. Mean arterial blood pressure (MAP) and heart rate (HR) were monitored (Model 870, PowerLab system, AD Instruments, Castle Hill, NSW, Australia) via the femoral artery, which was cannulated with polyethylene tubing (SP-31 Natsume Seisakusho Co., Ltd, Tokyo, Japan). The left common carotid artery was cannulated with a polyethylene tubing (SP-55 Natsume Seisakusho Co.) to serve as the arterial inflow cannula returning from the CPB circuit. A 16G cannula (Insyte BD Medical) was advanced through the right external jugular vein into the right atrium and served as a conduit for venous outflow to the circuit. The CPB circuit comprised of a venous reservoir (10 ml), a specially designed membranous oxygenator (Senko Medical Co., Ltd, Osaka, Japan), polyvinyl chloride tubing line (Senko Medical Co., Ltd), and a roller pump (Micro tube pump, MP-2000, Tokyo Rikakikai Co., Ltd, Tokyo, Japan) primed with 9 ml of Ringer's solution and 1 ml (1000 IU) of heparin. At the initiation of perfusion, the flow rate (6 ml/kg/min) was maintained for 240 min. In protocol 1, before the start of CPB rats randomly received the one of the following treatments: Sham group (cannulated for CPB without the pump); a saline treated CBP group (subcutaneous saline injection) and a ghrelin treated CPB group (one bolus ghrelin (150 μg/kg) subcutaneous injection). In protocol 1, CPB circulation was performed for 4 h without recovery from anesthesia. In two additional experiments, we examined if recovery from CPB within a clinically more relevant time frame for CPB procedures (2 h) altered several clinical markers of end organ damage, and if a second dose of ghrelin after CPB provided increased benefits. Following pretreatment (as for protocol 1) CPB was performed for 2 h, following which the tail artery and right jugular vein catheter were removed and the rats allowed to recover for a further 2 h (Protocol 2). Finally, in a separate group of rats, following pretreatment with ghrelin and CPB an additional dose of ghrelin (150 μg/kg) was administered as soon as the roller pump was stopped after 2 h CPB and the rats were allowed to recover for 2 h (Protocol 3). Arterial partial pressure of carbon dioxide (PaCO_2_), oxygen (PaO_2_), pH, hemoglobin (Hb) concentration, haematocrit (HCT) and electrolytes were measured at three defined time points (i-STAT Blood analyser, Abbott, NJ, USA).

### Plasma samples and specimen collection

Arterial blood samples (750 μl) were collected for blood gas measurements and subsequent molecular analyses at three defined time points; before CPB, and after 120 min and 240 min of CPB (Protocol 1–3). Tissue collection was performed in protocol 1 immediately after an overdose of isoflurane anesthesia. In each case, the brain, heart, lung, liver and left kidney were removed, washed with ice-cold saline and dissected into two parts. One part was fixed in 4% formaldehyde solution for histology and immunofluorescence study. The second part was frozen in liquid nitrogen and stored at −80°C for Western blotting.

### Organ damage analysis

Organ damage markers including lactate dehydrogenase (LDH), alanine aminotransaminase (ALT) and aspartate aminotransaminase (AST) were measured (DRI-CHEM 7000, Fujifilm, Kanagawa, Japan). These variables were measured by colorimetric qualitative analysis using commercially available FUJI DRI-CHEM slides.

### Measurement of plasma concentrations of catecholamines

Norepinephrine, epinephrine and dopamine levels were measured by using high-performance liquid chromatography with electrochemical detection as previously described (Takauchi et al., 1997).

### Inflammatory cytokines measurements

Plasma levels of tumor necrosis factor (TNF-α) and interleukin-6 (IL-6) were measured through enzyme-linked immunosorbent assay (ELISA), using commercial ultrasensitive Quantikine ELISA kits (TNF-α, 865.000.048; IL-6, R6000B, R&D Systems, Minneapolis, USA).

### Glutathione redox status assay

Abundance of reduced glutathione (GSH) and oxidized glutathione disulphide (GSSG) in plasma samples was determined using a Glutathione Assay Kit (# 703002, Cayman Chemical, USA) according to the manufacturer's instruction. Absorbance at 405 nm was measured using a microplate spectrophotometer (Polarstar Omega-6, BMG Labtech Ltd, Japan). Data collection and analysis were performed with the microplate data collection and analysis software Omega (BMG Labtech Ltd, Japan).

### Histological examination

Paraffin-embedded brain, heart, lung, liver and kidney samples were sectioned (5 μm), then stained with haematoxylin and eosin (H&E) to assess immune cell infiltration and tissue destruction. Collagen accumulations was evaluated by Azan-Mallory staining of kidney sections. Histological assessment of brain tissue injury was performed as previously described by Garman (2011). Histopathological analysis of the extent of injury in the heart, lung, liver and kidney were performed as previously described (Abdel-Salam et al., 2013). To make a preliminary examination of the extent of erythrocyte damage due to the CPB pump in 2 saline-treated rats a blood smear was prepared from blood samples at the three time points and the slides were visualized under light microscopy (40x) to quantify the proportion of crenated red blood cells or echinocytes (burr cells) and cell counts were averaged across 20 fields per sample.

### Western immunoblotting assay

Tissue samples including brain, heart, lung, liver and kidney were homogenized in ice-cold lysis buffer containing: 150 mM NaCl, 25 mM Tris-HCl (pH 7.5), 5 mM MgCl_2_ (pH 8.0) and 5% glycerol, 1% Triton X-100, complete EDTA free protease inhibitor cocktail (Roche, Indianapolis, IN). Protein concentrations in these homogenized samples were measured using the bicinchoninic acid (BCA) assay method. For Western blots, proteins were separated by SDS–PAGE and the following antibodies were used to quantify the myocardial protein expression levels: anti-CD-68 (1:1000, ab-31630), anti-nitrotyrosine (1:1000, NT, sc-32757), anti-interleukin-6 (1:1000, IL-6, ab-9324) and β-actin (1:1000, sc-47778) mouse polyclonal antibodies (Santa Cruz Biotechnology, USA) and anti-caspase-3 (1:1000, ab-9661S) rabbit polyclonal antibody (Abcam Laboratory, USA). For SDS-PAGE, 50 μg of protein was loaded on 10% sodium dodecyl sulfate-polyacrylamide gels (Bio-Rad, CA, USA), and subsequently transferred to nitrocellulose membranes. These membranes were then blocked with 5% nonfat dry milk and 3% BSA (Sigma, St. Louis, USA) in TBS-T (20 mM/l Tris, pH 7.6, 137 mM/l NaCl, and 0.05% Tween 20) at room temperature. After incubation with the primary antibodies, the bound antibody was visualized with an appropriate horseradish peroxidase (HRP)-coupled secondary antibody (1:10,000) (# 7076S; 7074S; Cell Signaling Technology, Inc. USA). Chemiluminescence signals were visualized using ATTO Chemi-luminescence Imaging System (Ez-Capture-II, ATTO Corporation, Tokyo, Japan) and were quantified using β-actin signal as a protein loading control (raw western blot images available as Supplemental Data Figure 2).

### Immunofluorescence staining

An immunofluorescence technique was used to determine the levels of macrophage by anti-CD-68 antibody in the brain, heart, lung, liver and kidney. Frozen sections were dried at room temperature for 10 min and rinsed 3 times in PBS for 5 min. Sections were blocked for 1 h in blocking buffer (1% BSA and 0.1% Triton x100 diluted in PBS). Sections were then incubated with the anti-CD-68 (1:1,000, ab-31630, Abcam) overnight at 4°C. The next day, the slides were washed 3 times for 5 min in PBS and incubated for 1 h room temperature with the goat anti-mouse (1:400, Alexa Fluor 568, Life Technologies) secondary antibody. Images were acquired using a fluorescence microscope (BX-53, Olympus, Tokyo, Japan). Positive cell counts per field were averaged across 20 fields per sample. The omission of primary antibodies in control experiments resulted in the expected absence of any cellular staining.

### *In situ* tunel apoptotic cell detection

We used *in situ* terminal deoxynucleotidyl transferase-mediated dUTP nick-end labeling (TUNEL) kit (MK 500, Takara, Shiga, Japan) to visualize apoptotic cells in the brain, heart, lungs, liver and kidney. Five micrometer thickness frozen sections were dried at room temperature for 10 min and rinsed 3 times in PBS for 5 min. Sections were incubated for 10 min with permeabilisation buffer. Sections were then incubated with the labeling reaction mixture (TdT enzyme 5 μl + labeling safe buffer 45 μl) for 90 min at 37°C in a humidified chamber. Slides were washed 3 times for 5 min in PBS to stop the reaction. TUNEL images were acquired using a fluorescence microscope (BX-53, Olympus, Tokyo, Japan). Positive cell counts per field were averaged across 20 fields per sample.

### Statistical analysis

All the data are presented as mean ± SEM. Two-way repeated measures ANOVA were used to compare time dependent changes between groups relative to the pre-CPB baseline, for the following parameters: LDL, ALT, AST, norepinephrine, epinephrine, dopamine, TNF-α, IL-6, GSH, and GSSG followed by Bonferroni's correction for multiple groups. Two-way repeated measures ANOVA were used to compare time dependent changes between groups for the three protocols for LDH, AST, ALT and IL-6 levels (protocol 1–3). Statistical analysis for treatment effects at given time points were examined with one-way ANOVA followed by Tukey's *post-hoc* test. Differences were considered statistically significant at a *p* < 0.05. Analyses were performed using Graph Pad Prism version 5.0 software (San Diego, CA, USA).

## Results

### CBP effects on haemodynamic variable and blood gas parameters

Table 1 shows the changes in haemodynamic variables and blood gas parameters including PaO_2_, PaCO_2_, Hb, and HCT in protocol 1 (Table 1). MAP, Hb, PaCO_2_, and HCT did not significantly change over the 4 h period in sham rats, but significantly declined during operation of the CPB (two-way ANOVA group x time interaction, *p* < 0.05; except PaCO_2_ interaction NS). The differences between saline and ghrelin treated CBP groups was not significant for any parameter other than HCT, which declined less in the ghrelin group (*p* = 0.034). As the shearing action of roller pumps is reported to contribute to haemolysis and deformation of erythrocytes during CPB (Möhnle et al., 2005), a preliminary observation suggests that indeed echinocyte numbers increased somewhat during CPB in saline-treated CPB rats (Supplemental Data Figures 1E,F). There was no significant difference between groups in the changes of HR, and changes in pH and electrolytes were minor, with all remaining within the physiological levels (two-way ANOVA group × time interaction for K+, *p* = 0.04, Table 1). Therefore, loss of erythrocytes is a contributing factor to the CPB injury, but this did not differ between saline and ghrelin treated rats.

**Table 1:** Hemodynamic variables, hemoglobin content, pH, electrolytes and blood gas partial pressures before and during four hours of CPB in rats pre-treated with saline or ghrelin and sham control rats (Protocol 1).

**Parameters**	**Group**	**Pre-CPB**	**CPB 120 min**	**CPB 240 min**
MAP (mm Hg)	Sham	108 ± 3	109 ± 4	98 ± 2
	CPB + Saline	111 ± 3	92 ± 6^**^^,^^##^	80 ± 4^**^^,^^###^
	CPB + Ghrelin	114 ± 4	87 ± 7^***^^,^^###^	88 ± 7^###^
HR (beats/min)	Sham	339 ± 9	343 ± 17	375 ± 19
	CPB + Saline	319 ± 14	346 ± 12	349 ± 24
	CPB + Ghrelin	313 ± 18	332 ± 22	364 ± 10^#^
PaO_2_ (mm Hg)	Sham	102 ± 13	87 ± 06	81 ± 03
	CPB + Saline	115 ± 07	144 ± 20^*^	167 ± 16^**^^,^^##^
	CPB + Ghrelin	96 ± 14	116 ± 17^*^	137 ± 13^#^
PaCO_2_ (mm Hg)	Sham	34 ± 2	33 ± 3	30 ± 3
	CPB + Saline	33 ± 2	30 ± 2	25 ± 2^###^
	CPB + Ghrelin	32 ± 2	29 ± 2	25 ± 2^###^
Hb (mg/dL)	Sham	13.7 ± 0.1	13.9 ± 0.2	13.5 ± 0.3
	CPB + Saline	14.2 ± 0.2	10.6 ± 0.2^***^^,^^###^	9.5 ± 0.2^***^^,^^###^
	CPB + Ghrelin	13.8 ± 0.2	11.1 ± 0.7^***^^,^^###^	10.6 ± 0.6^***^^,^^###^
HCT (%)	Sham	39.6 ± 0.81	41.0 ± 0.70	39.4 ± 0.92
	CPB + Saline	41.8 ± 0.66	31.2 ± 0.66^***^^,^^###^	28.0 ± 0.54^***^^,^^###^
	CPB + Ghrelin	40.6 ± 0.75	32.6 ± 2.07^***^^,^^###^	31.2 ± 1.79^***^^,^^###^^,^^a^
pH	Sham	7.53 ± 0.03	7.49 ± 0.03	7.49 ± 0.05
	CPB + Saline	7.52 ± 0.03	7.50 ± 0.04	7.51 ± 0.06
	CPB + Ghrelin	7.53 ± 0.04	7.48 ± 0.06^#^	7.45 ± 0.05^##^
Na (mEq/L)	Sham	136.20 ± 0.40	136.20 ± 0.60	136.50 ± 0.70
	CPB + Saline	136.40 ± 0.40	138.60 ± 0.90	141.20 ± 1.60^**^^,^^##^
	CPB + Ghrelin	137.00 ± 0.35	138.20 ± 0.82	139.40 ± 1.30
K (mEq/L)	Sham	3.96 ± 0.09	4.03 ± 0.11	4.16 ± 0.06
	CPB + Saline	4.02 ± 0.11	3.66 ± 0.05^#^	4.42 ± 0.14^#^
	CPB + Ghrelin	4.02 ± 0.08	3.85 ± 0.15	4.80 ± 0.18^**^^,^^###^

Hb, hemoglobin; HCT, haematocrit; HR, heart rate; MAP, mean arterial pressure; Na, sodium level; PaCO_2_, arterial partial pressure of carbon dioxide; PaO_2_, arterial partial pressure of oxygen; K, potassium level. The data shown are the mean ± SEM; N = 5 rats per group;

* p < 0.05,

** p < 0.01,

*** p < 0.001 vs. sham group;

# p < 0.05,

## p < 0.01,

### p < 0.001 vs. pre-CPB baseline,

a *p < 0.05 vs. saline-treated CPB group*.

### Ghrelin treatment improved end-organ damage markers

Several standard biochemical markers were utilized with differences in specificity for myocardial damage (LDH), liver damage (AST) and multi-organ injury (ALT) in all protocols. CPB induced an obvious increase in plasma LDH, AST, and ALT levels in a time-dependent manner compared to sham rats in protocol 1 (*p* < 0.001) (Figures 1B–D). Compared with the CPB saline treated rats, final plasma LDH levels were significantly reduced (240 min; *p* < 0.05), however AST and ALT levels were not as elevated in ghrelin-treated rats (Figures 1B–D).

### Ghrelin effect on catecholamine levels during CPB

**Figure 2:**
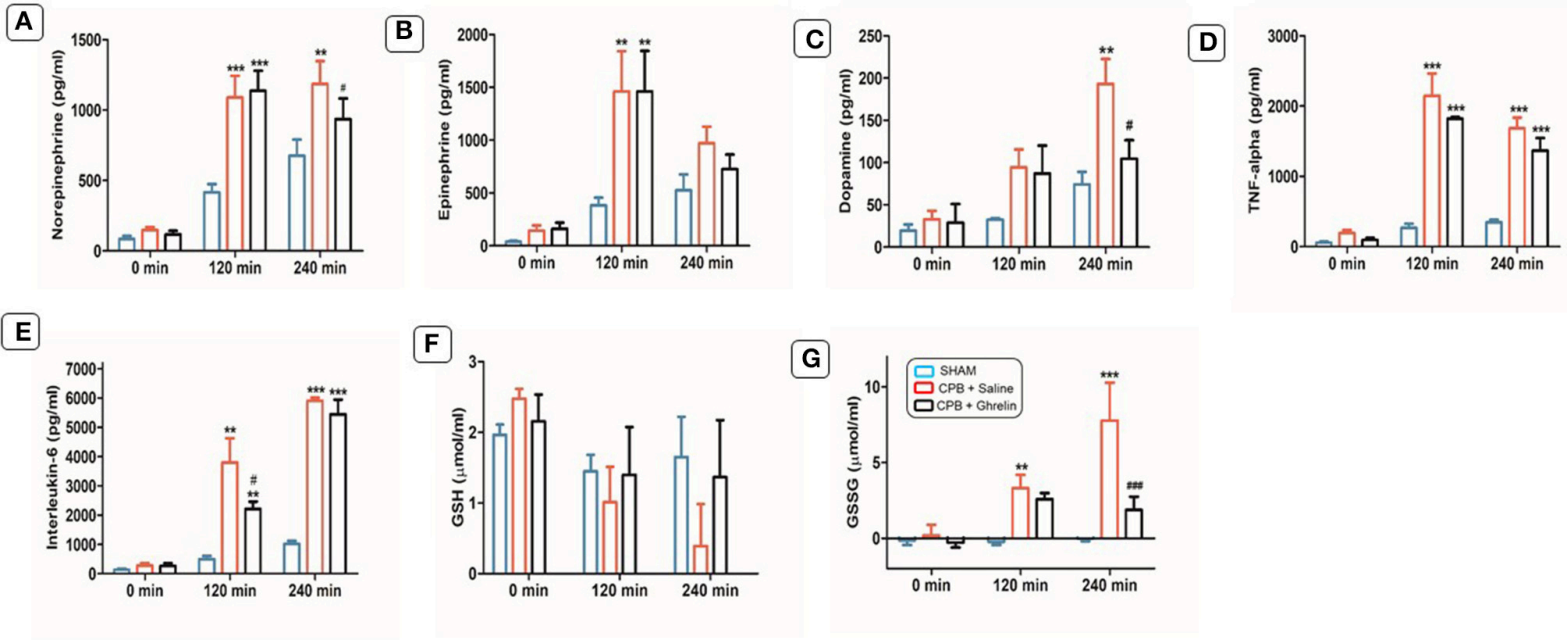
Ghrelin treatment partially improved plasma catecholamine, cytokine levels and the balance between reduced glutathione and oxidized glutathione in rats during CPB. Plasma levels of norepinephrine **(A)**, epinephrine **(B)**, dopamine **(C)**, TNF- α **(D)**, IL-6 **(E)**, total GSH **(F)**, and GSSG **(G)** before, and after 120 and 240 min of CPB. The data shown are the mean ± SEM; *N* = 5 rats per group. Statistical differences were determined by two-way ANOVA followed by Tukey's test or Student's unpaired *t*-test. **p < 0.01, ***p < 0.001 vs. sham group; ^#^*p* < 0.05, ^###^*p* < 0.001 vs. CPB saline group.

There were no significant differences in norepinephrine, epinephrine and dopamine levels in the basal state (0 min) between the three groups in protocol 1 (Figures 2A–C). In CPB groups, these catecholamine levels were significantly increased after 120 min (2.4-, 6.4- and 4.1-fold) and 240 min (1.7-, 2.0- and 5.0-fold) of CPB compared to sham rats. However, the increase in norepinephrine and dopamine in the ghrelin treated group after 240 min was significantly attenuated (0.4- and 1.4-fold) relative to the saline-treated CPB group. Similar results were observed for epinephrine (~50% lower), although statistical significance was not reached compared to CPB saline-treated group rats (Figures 2A–C).

### Ghrelin treatment did not alter systemic proinflammatory cytokine production

CPB elevated plasma levels of inflammatory cytokines (TNF-α: saline 8.0-fold and ghrelin 7.7-fold; IL-6: saline 5.3-fold and ghrelin 5.8-fold) levels significantly above that of sham group rats in protocol 1 (120 and 240 min; *p* < 0.001; Figures 2D,E). The attenuation of the increase in plasma TNF-α levels at 120 min and 240 min, was not significantly different from the saline treated CBP group (*p* > 0.05). However, there was a strong trend for ghrelin treatment to attenuate the early increase in IL-6 levels at 120 min of CPB (*p* = 0.052), although there was no difference from the saline-treated rats at 240 min (*p* = 0.771; Figures 2D,E).

### Ghrelin treatment improved glutathione content during CPB

In the saline treated CPB rat group in protocol 1, GSH levels were 3-fold lower after 240 min of CPB compared to sham group, while the GSH was not different from the baseline in ghrelin treated rats (*p* = 0.032; Figures 2F,G). GSSG levels - were nearly 9-fold higher in the CPB saline group compared to the sham group (*p* < 0.001). This increase in GSSG level during CPB was largely prevented by ghrelin pretreatment, indicating that ghrelin greatly attenuates oxidative stress induced by CPB (Figure 2G).

### Ghrelin partially improved brain proinflammatory cell infiltration and oxidative stress

**Figure 3:**
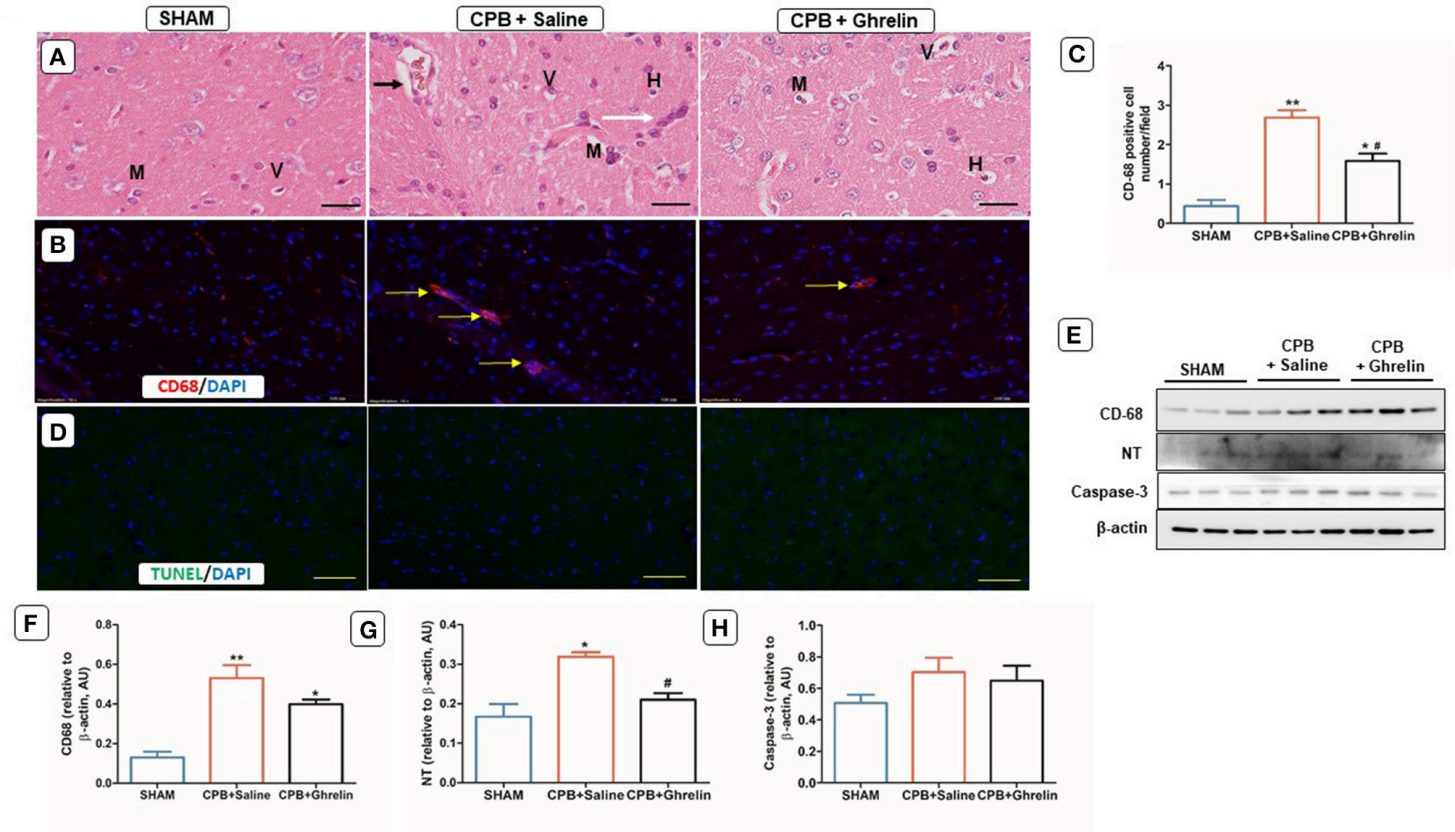
Ghrelin treatment partially reduced infiltration by leukocytes during CPB in the rat brain cerebral cortex. **(A)** H&E stained brain section of a sham rat showing normal features of primary immune cells; a CPB saline treated group (vehicle) rat showing focal inflammatory cell infiltration (white arrow), deeply stained dark nuclei (hyperchromatic cells) (H), multinucleated cells (M), vascular congestion (small black arrow), and vacuolated cells (V); a ghrelin treated CPB rat showing fewer infiltrating cells. **(B)** Immunofluorescence staining showed greater macrophage infiltration in the CPB saline group than in the ghrelin group. CD-68 positively-stained in microglial cells (evident in all groups) along with macrophages (yellow arrows) are shown by the red stain. **(C)** Quantitative measurement of CD-68 positive cell number/field across 20 fields. **(D)** *In situ* TUNEL-assay was used to assess the number of apoptotic cells (green stain)/field across 20 fields in the cerebral cortex. Nuclei were labeled with 4′, 6-diamidino-2-phenylindole (DAPI, blue). The merged image is presented in these panels. In all microscopy images the scale bar is 50 μm. **(E)** Protein expression levels of CD-68 (68 kDa), NT (72 kDa) and caspase-3 detected by Western blotting. β-actin (38 kDa) was used as an internal control. (F-H) Bar graph showing the densitometric analysis of the CD-68, NT and caspase-3 Western blots. The data shown are the mean ± SEM; *N* = 3 rats per group. Data were analyzed by a one-way ANOVA followed by Tukey's *post hoc* test. *p < 0.05, **p < 0.01 vs. sham group; ^#^*p* < 0.05 vs. CPB saline group.

Brain H&E staining showed that the sham group rats in protocol 1 had normal histology for the neurons in the cerebral cortex. CPB saline treated rats showed early signs of neuronal injury including inflammatory cell infiltration, hyperchromatic cells, mono-nucleated cells and vacuolated cells in the cerebral cortex (Figure 3A). In contrast, ghrelin treatment partly attenuated these early signs of neuronal injury (Figure 3A). Saline treated CPB group rats showed higher CD-68 macrophage accumulation (group *p* < 0.0001; Figures 3B,C) and protein expression when compared with sham rats (group *p* < 0.002; Figures 3E,F). In ghrelin treated CPB rats, a significantly lower number of macrophages were observed with microscopy (Tukey *p* = 0.005 vs. saline) but CD-68 protein expression was not significantly different from that in saline-treated rats (Tukey *p* = 0.162; Figures 3B,C,E,F). In addition, nitrosative stress marker (NT) protein expression was higher in the saline treated CPB group rats compared to sham rats and the ghrelin-treated CPB rats, which was not significantly different from the sham group (group *p* < 0.007, Tukey *p* = 0.03 saline vs. ghrelin Figures 3E,G). However, TUNEL positive apoptotic cells were not detected in regions of the cerebral cortex (Figure 3D) and protein levels of caspase-3 were not different between the three groups in protocol 1 (Figures 3D,E,H).

### Ghrelin did not reduce myocardial macrophage infiltration

**Figure 4:**
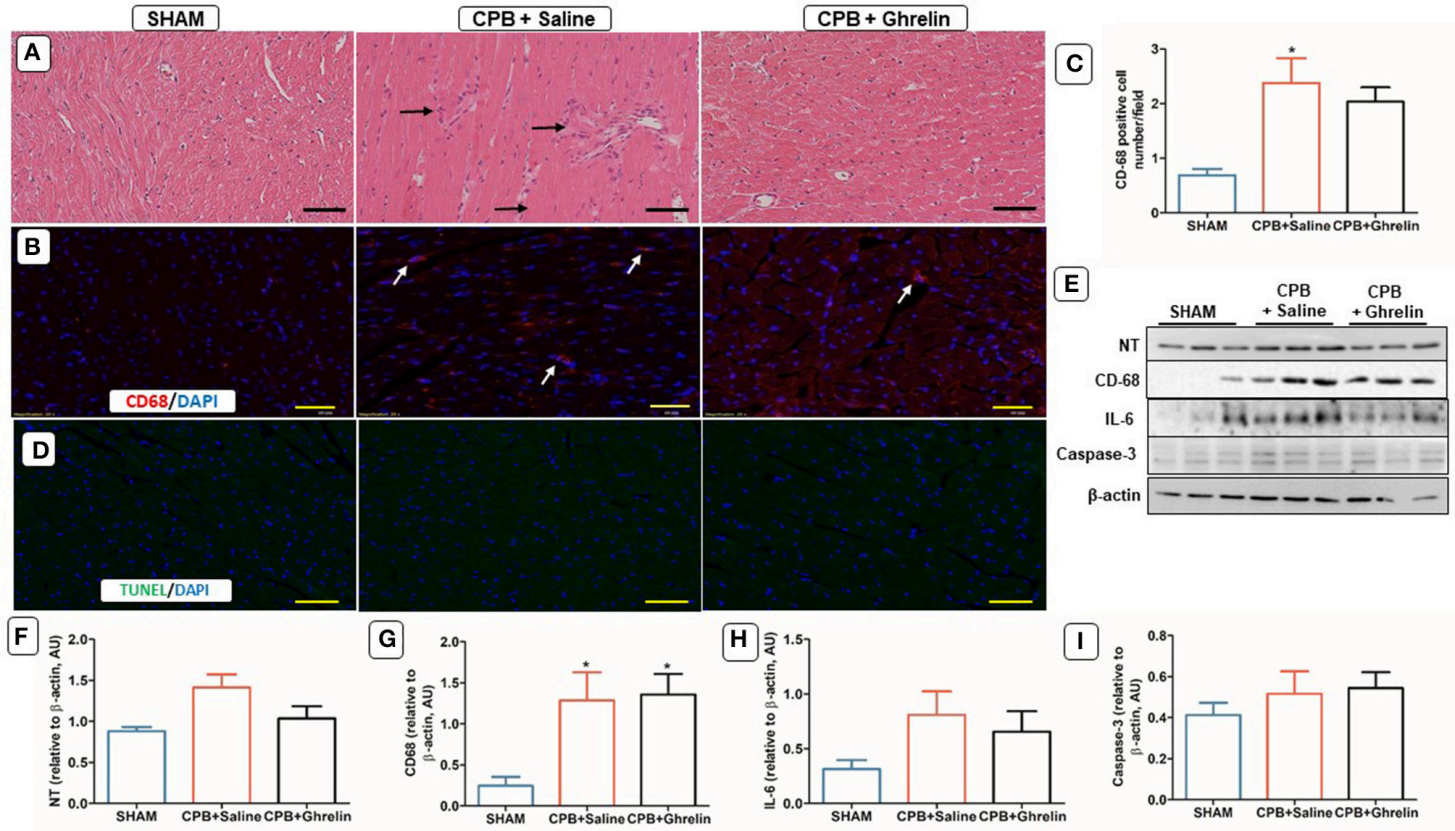
Ghrelin treatment did not reduce immune cell infiltration and oxidative stress due to CPB in the rat heart, but not IL-6 protein expression. **(A)** H&E stained heart section of a sham rat showing normal features of cardiomyocytes, blood vessels and endomysium; a CPB saline group rat showing focal accumulations of infiltrating immune cells (black arrows) and degenerating myocardial cells; a ghrelin treated rat showing reduced focal accumulations and a marginal number of degenerating myocardial cells. **(B)** Immunofluorescence staining was used to access the levels of CD-68 macrophages (red stain, white arrows) positively-stained in cardiomyocytes. **(C)** Quantitative measurement of CD-68 positive cell number/field across 20 fields. **(D)** *In situ* TUNEL-assay was used to access the apoptotic cell number (green stain)/field across 20 fields in the myocardium. Nuclei were labeled with 4′, 6-diamidino-2-phenylindole (DAPI, blue). The merged image is presented in this figure. In all images scale bar is 50 μm. **(E)** Myocardial protein expression levels of NT, CD-68, IL-6, and caspase-3 was detected by Western blotting. β-actin was used as an internal control. **(F–I)** Bar graphs showing the densitometric analysis of the NT, CD-68, and IL-6 Western blots. The data shown are the mean ± SEM; *N* = 3 rats per group. Data were analyzed by a one-way ANOVA followed by Tukey's *post hoc* test. *p < 0.05 vs. sham group.

H&E and immunofluorescence stained sections revealed a significant upregulation of infiltrating cells in the myocardium in both saline and ghrelin treated rats after 4 h of CPB relative to the sham rats (Figures 4A–C). In addition, the Western blotting confirmed that there were significantly elevated protein levels of CD-68 (group *p* = 0.037) in the myocardium of both CPB groups in protocol 1, but cardiac nitrotyrosine (group *p* = 0.063 trend only) and IL-6 expression (group *p* = 0.193) were not different from sham rats (*p* = NS; Figures 4E–H). There were no significant differences in caspase-3 protein expression (*p* = 0.547) and apoptotic cells were not observed in the myocardium (Figures 4D,E,I).

### Ghrelin treatment partly attenuated CPB lung inflammation and oxidative stress

**Figure 5:**
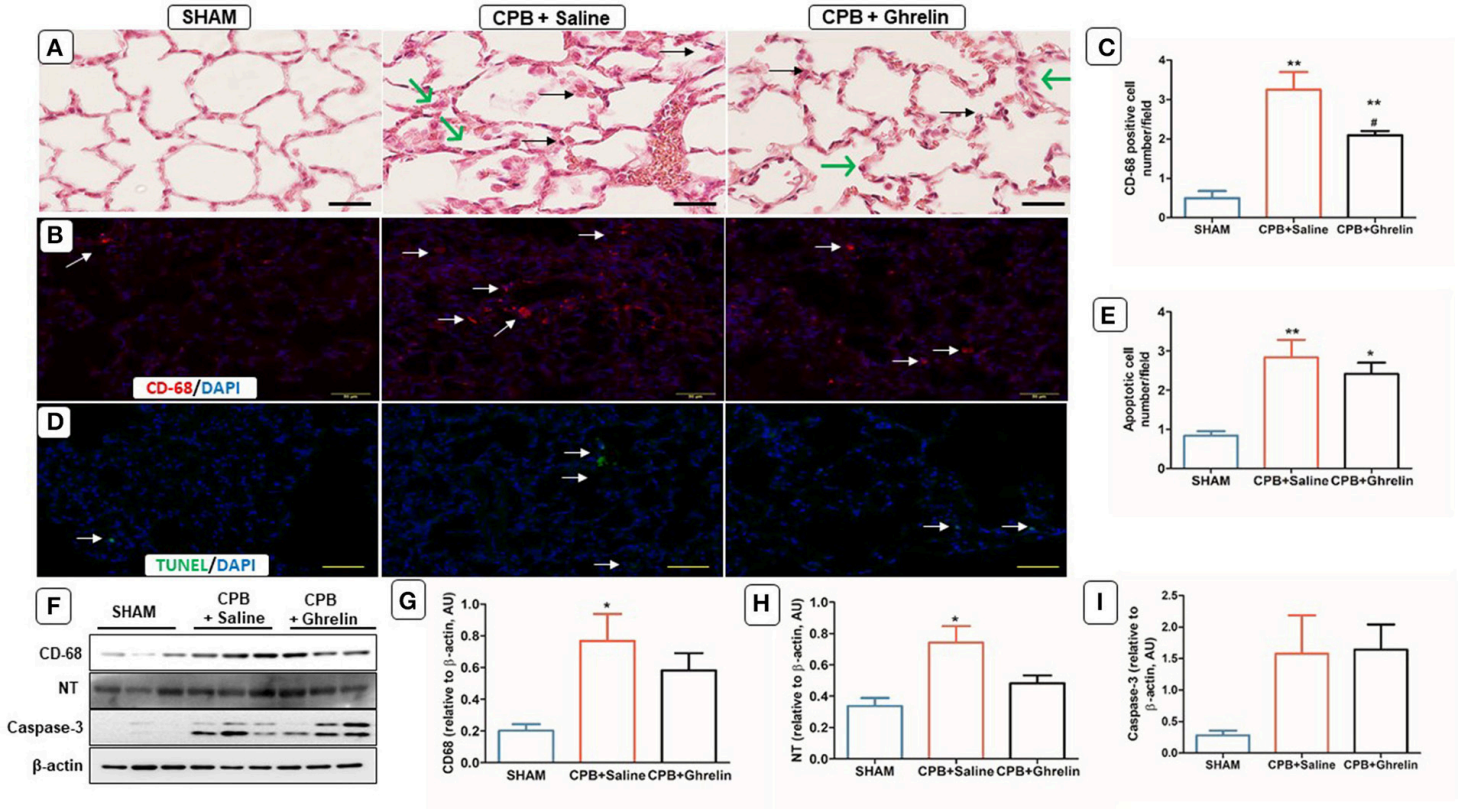
Ghrelin treatment improved inflammation, oxidative stress but not apoptosis in rat lungs following CPB. **(A)** H&E stained lung section of a sham rat showing normal features of alveolar capillary membrane; a CPB saline group rat showing inflammatory cell infiltration (black arrows) and thickening of alveolar capillary membrane (green arrows); a ghrelin treated rat showing reduced inflammatory cell infiltration (black arrows) but thickening of the alveolar capillary membrane (green arrows). **(B)** Immunofluorescence staining was used to access the levels of CD-68 macrophages (red stain; white arrow) positively-stained in alveolar capillary membrane. **(C)** Quantitative measurement of CD-68 positive cell per 20 fields. **(D)** *In situ* TUNEL-assay was used to access the apoptotic cells per 20 fields (green stain; white arrow) in the lungs. Nuclei were labeled with 4', 6-diamidino-2-phenylindole (DAPI, blue). The merged image is presented in this figure. In all images scale bar is 50 μm. **(E)** Quantitative measurement of apoptotic cell number/field across 20 fields. **(F)** Protein expression levels of CD-68, NT and caspase-3 detected by Western blotting. β-actin was used as an internal control. **(G–I)** Bar graph showing the densitometric analysis of the CD-68, NT and caspase-3 Western blots. The data shown are the mean ± SEM; *N* = 3 rats per group. Data were analyzed by a one-way ANOVA followed by Tukey's *post hoc* test. *p < 0.05, **p < 0.01 vs. sham group; ^#^*p* < 0.05 vs. CPB saline group.

H&E stained sections showed normal lung tissue morphology in sham rats (Figure 5A). There was pronounced thickening of alveolar capillary membranes and inflammatory cell infiltration in saline treated CBP group rats. In contrast, ghrelin treatment did not alter membrane thickening, but attenuated the accumulation of infiltrating cells compared to saline-treated CPB rats (Figure 5A). Immunofluorescence staining showed greater CD-68 positive macrophage accumulation in the lungs of saline treated CPB rats than in ghrelin treated CPB rats (Tukey *p* < 0.05; Figures 5B,C). Although the Western blot results showed significant elevation in protein levels of CD68 (group *p* = 0.039) and nitrotyrosine (*p* = 0.022) in CBP rat groups, neither protein was significantly elevated relative to the shams following ghrelin treatment (Tukey post hoc test; Figures 5F–H). In contrast to the myocardium, the number of TUNEL-apoptotic cells and caspase-3 protein expression in the lungs was markedly increased by CPB in both saline treated and ghrelin treated CPB rats compared to sham rats, but this did not reach statistical significance for caspase-3 protein (apoptotic cell number *p* = 0.004 and *p* = 0.016) (Figures 5D–F,I).

### Ghrelin treatment partially reduced liver injury but not macrophage infiltration

**Figure 6:**
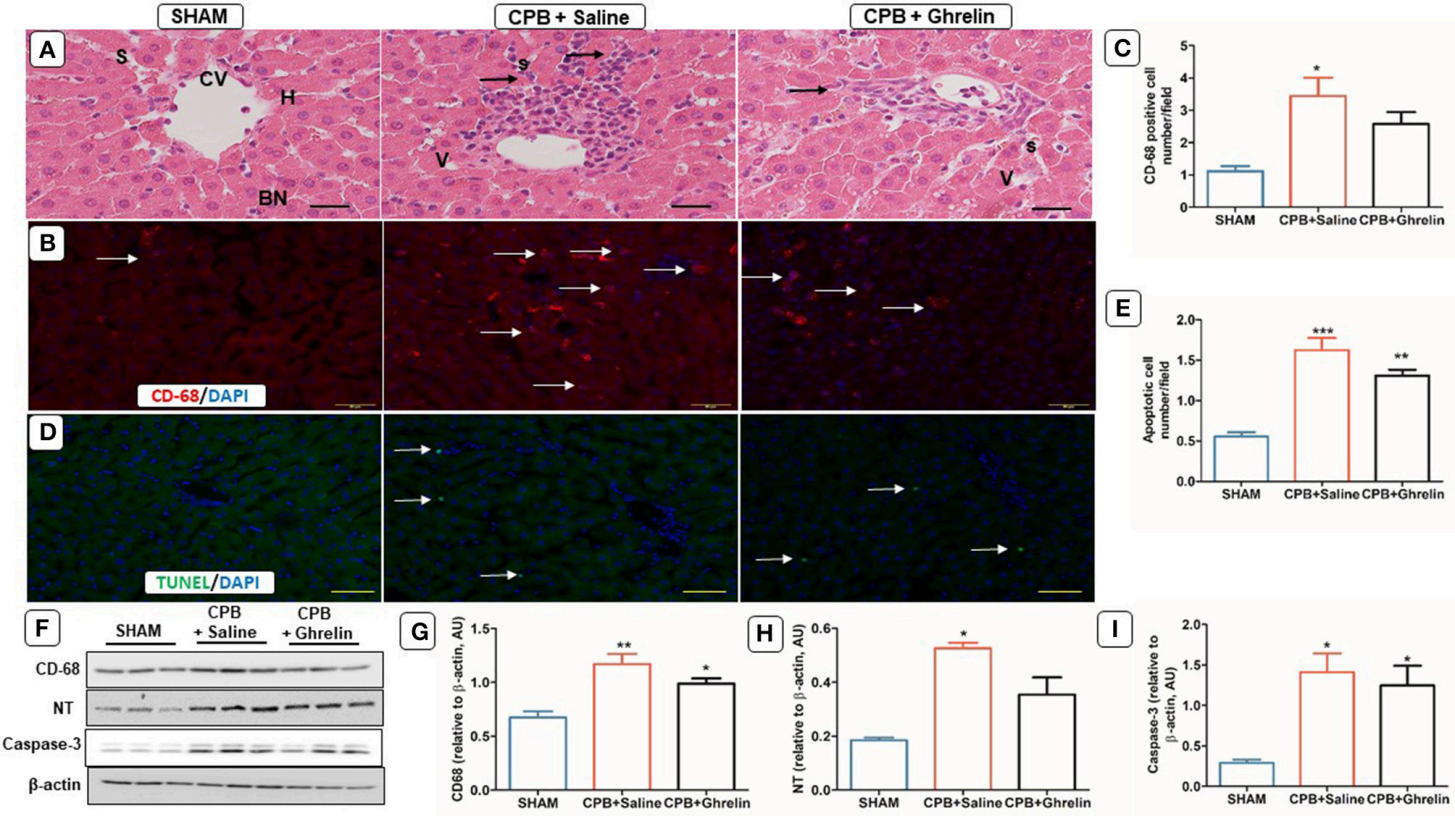
Ghrelin treatment improved liver inflammation, oxidative stress but not apoptosis in rats following CPB. **(A)** H&E stained liver section of a sham rat showing normal features of central vein (CV), hepatocytes (H), sinusoids (S) and bi-nucleated cells (BN); a CPB rat showing increased mononuclear cell infiltration (black arrows) and vacuolated cells (V); a ghrelin treated rat showing fewer V and infiltrating cells (black arrows). **(B)** Immunofluorescence staining was used to access the levels of CD-68 macrophage (red stain; white arrows) positively-staining in the liver. In all images the scale bar is 50 μm. **(C)** Quantitative measurement of CD-68 positive cell number/field across 20 fields. **(D)** *In situ* TUNEL-assay was used to assess the apoptotic cell number (green stain; white arrow)/field across 20 fields in liver. Nuclei were labeled with 4′, 6-diamidino-2-phenylindole (DAPI, blue). The merged image is presented in this figure. In all images scale bar is 50 μm. **(E)** Quantitative measurement of apoptotic cell number/field across 20 fields. **(F)** Protein expression levels of CD-68, NT and caspase-3 detected by Western blotting. β-actin was used as an internal control. **(G–I)** Bar graph showing the densitometric analysis of the CD-68 and NT caspase-3 Western blots. The data shown are the mean ± SEM; *N* = 3 rats per group. Data were analyzed by a one-way ANOVA followed by Tukey's *post hoc* test. *p < 0.05, **p < 0.01, ***p < 0.001 vs. sham group.

Histological examination of liver sections showed deranged liver architecture, vacuolar degeneration, focal necrosis, and obvious infiltration of inflammatory cells in saline treated CPB rats compared to sham rats (Figure 6A). In contrast, ghrelin treated rats showed improved architecture and slightly less necrosis than CPB group rats (Figure 6A). Saline treated CPB rats showed increased CD-68 macrophage accumulation when compared to sham rats (group *p* = 0.017, Tukey *p* = 0.014 sham vs. saline Figures 6B,C). Ghrelin treatment did not reduce the accumulation of CD-68 cells to a significant extent compared with saline treated CPB rats, but it was reduced such that cell numbers were not significantly different from the sham group (Tukey *p* = 0.090; Figures 6B,C). In addition, protein expression of CD-68 (group *p* = 0.007) and NT (*p* = 0.012) were increased by CPB treatment relative to sham rats. Similarly, ghrelin treatment did not attenuate CD-68 or NT expression when compared to saline treated CPB group rats (Tukey *post hoc* test; Figures 6F–H). TUNEL staining revealed a significant upregulation of apoptotic cell number and caspase-3 protein expression in CPB treated rats compared to sham rats (group *p* = 0.014). Conversely, ghrelin treated did not reduce the number of apoptotic cells (Tukey *p* = 0.120) and caspase-3 protein expression compared to saline-treated CPB rats (Tukey *p* = 0.830; Figures 6D–F,I).

### Ghrelin improved renal macrophage infiltration

**Figure 7:**
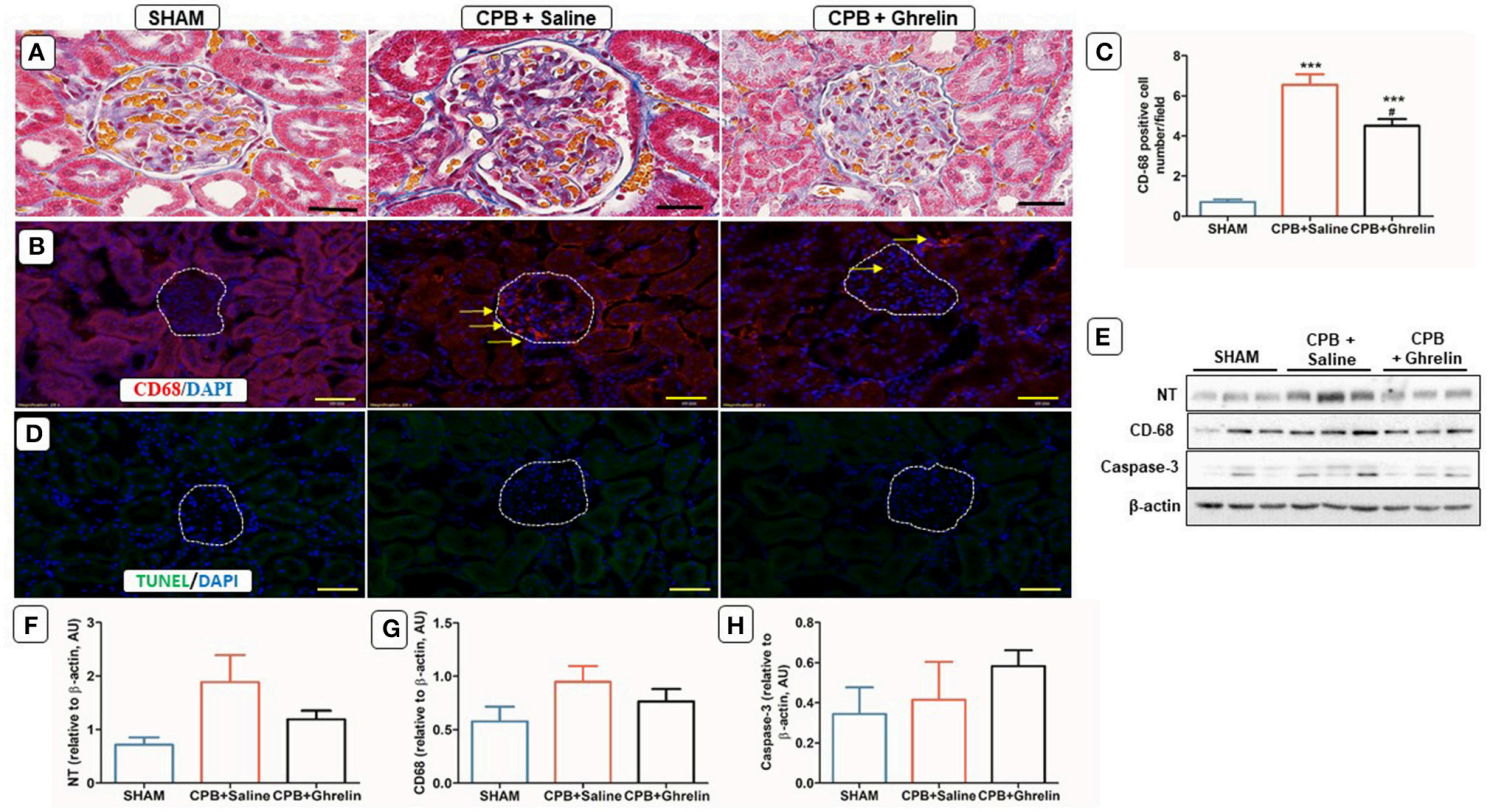
Ghrelin treatment improved renal inflammation, but not apoptosis in rats following CPB. **(A)** Azan-Mallory stained kidney section of a sham rat showing a normal glomerular membrane, capsular and tubular capillaries; a CPB rat showing the presence of collagen accumulations (blue staining) surrounding the tubules, inflammatory cell infiltration within the glomerulus and increased capsular space; a ghrelin treated rat showing reduced cell infiltration and capsular space (40x). **(B)** Immunofluorescence staining was used to access the levels of renal cortical CD-68 (red stain; yellow arrows) positively-stained cells. **(C)** Quantitative measurement of CD-68 positive cell per 20 fields. **(D)** *In situ* TUNEL-assay showed the absence of apoptotic cells (green stain) in the glomeruli. Nuclei were labeled with 4', 6-diamidino-2-phenylindole (DAPI, blue). The merged image is presented in this figure. In all images scale bar is 50 μm. **(E)** Protein expression levels of NT, CD-68 and caspase-3 detected by Western blotting. β-actin was used as an internal control. **(F–H)** Bar graph showing the densitometric analysis of the NT, CD-68 and caspase-3 Western blots. The data shown are the mean ± SEM; *N* = 3 rats per group. Data were analyzed by a one-way ANOVA followed by Tukey's *post hoc* test. ***p < 0.001 vs. sham group; ^#^*p* < 0.05 vs. CPB saline group.

The kidneys of CPB rats were characterized by mild glomerulosclerosis, as evidenced by mesangial expansion, and the presence of inflammatory infiltration within the glomeruli and tubules. Ghrelin treatment appeared to reduce infiltration and membrane thickening when compared to CPB rats (Figure 7A). CPB treatment increased cortical CD-68 macrophage cell number (group *p* < 0.0001), but was significantly attenuated by ghrelin treatment (Tukey *p* < 0.009 vs. saline group; Figures 7B,C). However, the expression levels of CD-68 protein and nitrotyrosine did not differ significantly between groups (*p* = 0.268 and *p* = 0.100; Figures 7E–G). This difference between immunohistofluorescent positive cell counts and protein expression most likely reflects the fact that the former is restricted to glomeruli whereas Western blotting was performed with renal cortical tissue. TUNEL-apoptotic cells were not detected in the cortical regions of the kidney and there was no difference in caspase-3 protein expression between the groups (*p* = NS; Figures 7D,E,H).

### The effects of repeated ghrelin delivery on clinical markers following 2 h of CPB

As shown in Supplemental Data Figures 1A–C, two doses of ghrelin administration significantly decreased LDH levels compared to the single pre-CPB dose group rats at the end of CPB (120 min; *p* = 0.003), but this difference between ghrelin groups declined during the 2 h recovery time. Notably, in rats given one bolus before CPB (protocol 2) and rats given the second bolus of ghrelin post-CPB (protocol 3), final LDH at 240 min was much reduced compared to ghrelin treated rats in protocol 1 (protocol 1 ghrelin treated CPB 1432 ± 125 U/l *N* = 5, protocol 2 CPB 874 ± 311 U/l N = 4 and protocol 3 CPB 416 ± 127 U/l N = 3; Tukey *p* < 0.001). Furthermore, final LDH in protocol 3 was not significantly different from the sham group (sham 216 ± 37 U/l, Tukey *post hoc* test). However, there was no significant difference in AST at the end of CPB or 2 h following CPB between protocols 2 and 3 (protocol 1 ghrelin treated CPB 121 ± 5 U/l *N* = 5, protocol 2 CPB 146 ± 21 U/l *N* = 4 and protocol 3 CPB 128 ± 12 U/l *N* = 3 Tukey post hoc test; Supplemental Data Figure 1B), whereas final ALT only was moderately elevated in protocol 2 and 3 groups compared to ghrelin treated CPB in protocol 1 (protocol 2 CPB 80 ± 22 U/l *N* = 3 and protocol 3 CPB 72 ± 12 U/l *N* = 3, *p* = 0.001 and *p* = 0.013 vs. protocol 1 ghrelin treated CPB 42 ± 1 U/l *N* = 5; Supplemental Data Figure 1C). Therefore, shorter CPB duration and a second bolus of ghrelin after CPB further greatly reduced the increase in LDH relative to more the prolonged CPB protocol and the single pretreatment dose of ghrelin. IL-6 plasma levels in the single dose ghrelin treated rats (protocol 2, 6,524 ± 431 pg/ml *N* = 4, Tukey post hoc test *p* < 0.001) and double dose ghrelin treated rats (protocol 3, 4,376 ± 1,095 pg/ml *N* = 3, Tukey post hoc test *p* = 0.074) were triple and double respectively (Supplemental Data Figure 1D), the levels seen at midpoint of CPB in protocol 1 for ghrelin treated rats (120 min, 2210 ± 254 pg/ml, Figure 2E). However, in both ghrelin treated groups that were exposed to the shorter CPB duration the final plasma IL-6 levels were similar to ghrelin treated rats in protocol 1 at 240 min (Tukey post hoc test NS; Supplemental Data Figure 1D and Figure 2E, protocol 1 ghrelin treated CPB 5447 ± 498 pg/ml *N* = 5, protocol 2 ghrelin CPB 4,336 ± 1,127 pg/ml *N* = 4 and protocol 3 ghrelin CPB 3,491 ± 1519 pg/ml *N* = 3). This finding suggests IL-6 increases were largely dependent on CPB duration, peaking soon after CPB, and were independent of ghrelin treatment *per se*.

## Discussion

**Figure 8:**
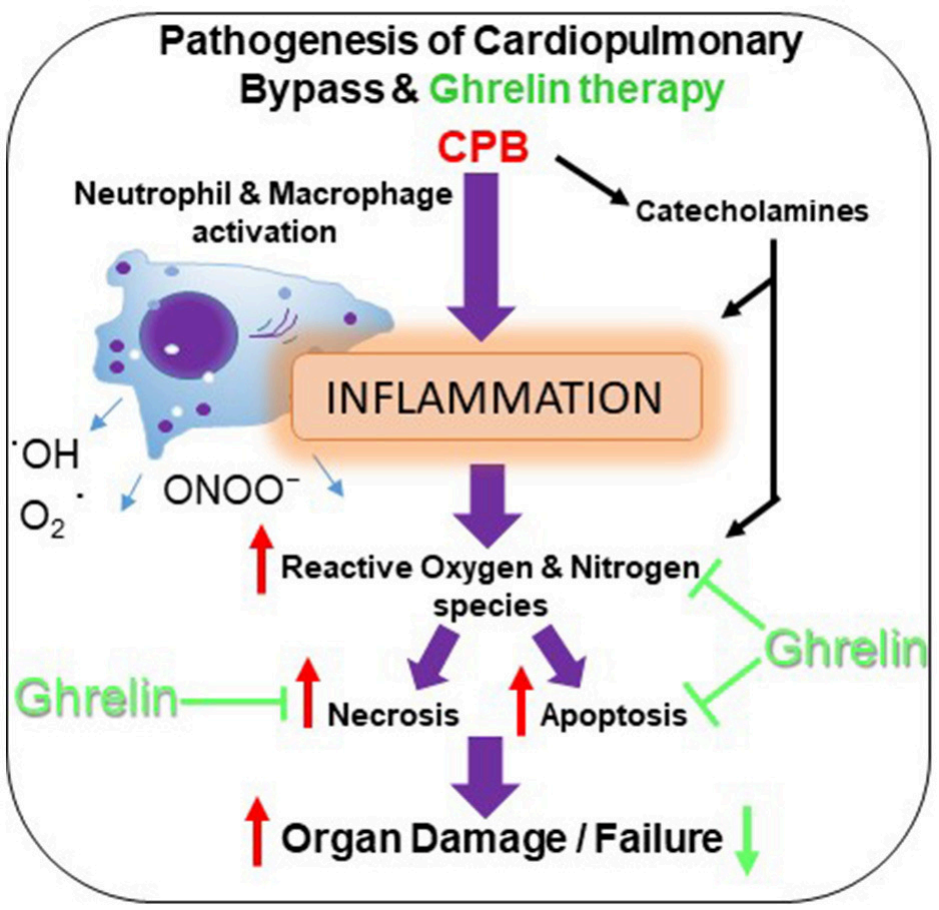
Schematic representation showing the beneficial effects of ghrelin treatment on CPB-induced organ injury.

In the present study, we investigated whether a bolus of ghrelin administered at the time of CPB ameliorated the end organ damage induced in multiple organs by CPB in anesthetized rats and if this involves suppression of systemic and tissue levels of cytokines, leukocyte infiltration, oxidant/antioxidant imbalance and oxidative damage. We found the main effects of ghrelin treatment in our model of prolonged CPB (4 h duration) were that it greatly suppressed the imbalance of oxidants to antioxidants and consequent end organ damage in the brain, lung and liver. Nonetheless, ghrelin had little effect on plasma norepinephrine level and only partially suppressed the infiltration by macrophages to varying degrees in different organs. Ghrelin treatment did not attenuate the production of the pro-inflammatory cytokines IL-6 and TNF-α as anticipated, suggesting that such anti-inflammatory actions of ghrelin might be dependent on the activity of specific signaling mechanisms. Further, the low levels of caspase-3 activation and expression of TUNEL-positive apoptotic cells, primarily in the lungs and liver, were not really affected by ghrelin in this study. Cao et al. (2013), reported large reductions in myocardial damage following ghrelin treatment at the time of CPB, and greater reductions in the elevated production of IL-6 and TNF-α in the myocardium compared to plasma levels (assessed 4 h after the 1 h of CPB). Furthermore, the proportion of apoptotic cells in the myocardium in both vehicle and ghrelin treated rats in that study was much higher than this study. To consider the possibility that CPB injury might be enhanced following recovery from anesthesia we investigated the effect of ghrelin given at the time of CPB in rats where CPB was limited to 2 h duration and rats were allowed to recover for a further 2 h before collecting final plasma and tissue samples (comparable to the Cao et al., 2013 study). Further, we also attempted to determine if the dose/timing of ghrelin delivery was a factor contributing to differences between studies. Again these findings showed that plasma IL-6 and TNF-α were not influenced by the shorter CPB and combined recovery period or administration of a second ghrelin bolus immediately after CPB. Possible explanations for these differences between this study and Cao et al. (2013) are given further consideration below. Nonetheless, this study utilizing an anesthetized rat model reaffirms that CPB-induced injury is widespread, but greatest in the lungs and liver at the end of CPB. It also shows that while apoptosis in the myocardium, brain and kidney was very low despite a prolonged period of CPB, ghrelin partially reduced infiltration of various leukocyte cell types, especially macrophages, and nitrosative stress induced tissue modifications in the brain, lung and liver. Furthermore, as ghrelin treatment facilitated the maintenance of oxidant/anti-oxidant balance, as demonstrated by the maintenance of reduced glutathione (GSH) production and decreased production of oxidized glutathione (GSSG), throughout the prolonged duration of CPB in this study (protocol 1). This suggests that ghrelin's actions might counter the multiple sources of oxidative stress that are likely to be induced by CPB, and not just nitrotyrosine. Consistent with the study of Cao et al. (2013), we found that ghrelin reduced end organ damage associated with necrosis even though inflammation and oxidative/nitrosative stress are not completely attenuated (Figure 8).

In this study one of the main findings was that pre-treatment with ghrelin partially reduced the infiltration of macrophages in multiple organ systems and subsequent oxidative-nitrosative stress that was observed by histology and immunofluorescence techniques. In addition, we found that ghrelin essentially normalized standard clinical indicators of end organ damage (ALT and AST) to sham levels even during prolonged CPB. Further, although the level of another clinical index of end organ damage, LDH, increased over the course of CPB in protocol 1, and was not reduced by ghrelin in that experiment (Figure 1B) we showed that an additional post-CPB dose of ghrelin after CPB greatly reduced LDH in protocol 3, to nearly sham group level. CPB has been shown many times, to induce inflammation that in turn leads to dysfunction in multiple organ systems, including the cardiovascular, pulmonary, hepatic, renal and central nervous systems (Chello et al., 2006; Gottesman and Wityk, 2006; Gottesman et al., 2008; Heusch et al., 2012; Fujii et al., 2015, 2016). This increase in LDH with time is most likely attributable to haemolysis resulting from the shear forces associated with the action of the roller pump used in this study, as previously reported (Möhnle et al., 2005). The release of Hb could conceivably compound systemic oxidative stress during CPB. Nonetheless, we also found that ghrelin slightly, but significantly attenuated the decline in HCT over time, but the reason for this remains unclear (Table 1). It is well established that CPB induces a systemic inflammatory response that increase the production of reactive oxygen species (ROS) and reactive nitrogen species to cause end-organ damage through apoptosis and necrosis (Goebel et al., 2008; Li et al., 2012; Zakkar et al., 2015). Following surgery various indicators of severe oxidative stress are often reported to be associated with acute lung injury in patients, including chlorotyrosine, nitrotyrosine, and orthotyrosine (Lamb et al., 1999; Callister et al., 2006). Protein tyrosine nitration is a routinely used biomarker for ^·^NO-dependent oxidative stress (Radi, 2004). In this study, we found that this oxidative modification of proteins was greatest in the brain, liver and lung, but the increase in expression was lower in the heart and kidney. Notably, even though macrophages had infiltrated the liver and lung during the course of CPB, ghrelin was able to nearly completely prevent the increase in protein tyrosine nitration in the lung and attenuate this increase by half in the liver (Figures 5, 6). Activated macrophages are the leading source of peroxynitrite generation leading to nitrotyrosine formation (Ahsan, 2013). Therefore, it is reasonable to suggest that improvement in the end-organ injury by ghrelin pre-treatment is through the suppression of the activation of the macrophages that infiltrate various organs, and subsequent reduction in the elevation of oxidative stress in these tissues.

Cao et al. (2013) first showed that ghrelin pre-treatment reduced CPB injury in the heart of rats following 1 h of CPB. Although a similar system was used in both studies, those authors showed more pronounced myocardial injury than this study, with greater myocardial apoptosis, a more pronounced increase in LDH and a six-fold increase in cardiac TnI level. The reasons for the differences in the extent of myocardial injury are not clear, but an important difference was that our CPB protocol 1 was more prolonged and the rats were entirely under anesthesia. Cao et al. (2013) performed 1 h of CPB, then rats were weaned-off of the CPB and allowed recovery of the rats before re-inducting anesthesia to measure cardiac function invasively and collect plasma and tissue samples 4 h post CPB. Even though those authors showed that more cells became apoptotic in a similar period of time as this study, the IL-6 and TNF-α plasma concentrations were ten-fold less than our measurements in protocol 1. In support of the idea that the cytokines are perhaps directly related to CPB duration is the fact that IL-6 levels were lower in the ghrelin treated rats exposed to 2 h of CPB compared to 4 h in this study.

Our main findings and those of others in the field of immunology lead us to believe that there is an important role for ghrelin in modulating leukocyte activation and oxidative stress, but there are important differences between studies in regard to the regulation of local pro-inflammatory responses. There is strong evidence that the anti-inflammatory properties of ghrelin are also mediated through modulation of autonomic neural activity. Lymphocytes and monocytes, which are important sources of pro-inflammatory cytokines, express ghrelin and its receptor GHS-R. Dixit et al. (2004) previously showed that ghrelin administration suppressed inflammation and ROS generation in these leukocyte lines. Ghrelin suppressed the release of IL-6 and TNF-α in the peritoneal cavity (Wu et al., 2007a) and the lungs (Wu et al., 2007b) in a sepsis model and in the brain in a focal cerebral ischaemia model (Cheyuo et al., 2011) in rats through a mechanism that requires intact vagus nerves. Furthermore, the same researchers showed that in addition to suppression of peripheral macrophage production of cytokines via the stimulation of vagal activity, which results from central GHS-R activation by ghrelin, this hormone is able to reduce peripheral release of TNF-α by a direct sympatho-inhibitory effect (Wu et al., 2007c). In our study, we observed that ghrelin partially reduced organ infiltration by macrophages (CD68 positive immunostaining and relative protein expression), but did not prevent the release of pro-inflammatory cytokines. This raises the question why then did ghrelin not reduce IL-6 and TNF-α during CPB in this study?

It has been established that the pro-inflammatory response induced by CPB is initiated as result of blood contact with the surface of the extracorporeal tubing, haemodilution from red blood cell destruction and subsequent activation of complement factors and vascular adhesion molecules (Kotani et al., 2000; Gao et al., 2015). Most clinical and experimental studies report that pro-inflammatory cytokines in plasma reach a peak during CPB surgery (generally 2 h duration) and remain elevated for 3–6 h (McBride et al., 1995; de Mendonça-Filho et al., 2006; Cao et al., 2013). CPB evokes increases in plasma levels of IL-8, IL-6, and TNF-α, and even greater increases in both gene expression and cytokine levels in the alveolar macrophages (Kotani et al., 2000). These authors have suggested that infiltrating macrophages in the lungs play the greater role in the pro-inflammatory response associated with CPB, which typically results in widespread distal airway epithelial and endothelial injury. For this reason, we examined CD68-positive macrophage roles in CPB injury in this study. However, Cao et al. (2013) has shown that activated neutrophils contribute to myocardial injury through an increase in myeloperoxidase activity. Therefore, it seems that in this study at least, acute delivery of ghrelin pre- or post-CPB was not able to prevent the increase in IL-6 and TNF-α caused by blood contact with the extracorporeal circuit, even though it has been demonstrated in multiple studies that macrophages express GHS-R and that ghrelin activates autonomic reflexes that are capable of suppressing pro-inflammatory cascades involving IL-6 and TNF-α (Wu et al., 2007a,b; Cheyuo et al., 2011). Whether the reason for the differences between studies is dependent on the route of administration of ghrelin and the time taken for ghrelin to suppress IL-8 formation during CPB remains to be determined.

In conclusion, the present study shows that in the rat model of CPB-induced systemic inflammation, pre-CPB and post-CPB bolus administration of ghrelin decreased the injury in multiple organs through reduction of inflammatory response and oxidative stress. Therefore, we consider that bolus delivery of ghrelin might provide an effective approach for reducing widespread CPB-induced organ injury. In future experiments, it will be important to investigate how ghrelin pre-treatment affects cardiovascular function and organ repair processes following recovery from CPB.

### Study limitations

Animals in which the Hb levels declined to less than 8 g/dL at any point were excluded from the study as they were considered to be non-physiological conditions for recovery from CPB. Ghrelin was delivered as a single bolus by subcutaneous injection. We therefore cannot rule out the small possibility that during the course of CPB that ghrelin taken up by the circulation might have been removed by the extracorporeal circuit due to residual charge on its contact surfaces. In addition, we must acknowledge that only 4 and 3 rats respectively were used in protocols 2 and 3. Furthermore, in this study we did not examine how the combination of ghrelin and steroid administration would have affected CPB-induced injury. As steroids such as methylprednisolone are often used in CPB, and have been shown to reduce IL-8 production during CPB (Gormley et al., 2003), it is possible that combination therapy might have added benefits. This remains to be investigated in future studies.

## Author contributions

VS and JP designed the experiments with consultation of MS and drafted the manuscript. VS conducted all the experiments and conducted the statistical analyses. YF and HT contributed to setup and conduct of the CPB protocol. TA performed the measurements of the catecholamines. HH, KK, and ET provided intellectual contributions. All authors approved the final version of the manuscript.

### Conflict of interest statement

The authors declare that the research was conducted in the absence of any commercial or financial relationships that could be construed as a potential conflict of interest.

## Supplementary material

Supplemental Data Figure 1 Effects of single dose ghrelin (protocol 2) versus a double dose of ghrelin (protocol 3) on LDH **(A)**, ALT **(B)**, AST **(C)**, and IL-6 **(D)** before CPB, and after 120 min of CPB and a further 2 h after recovery from CPB (240 min). **(E)** Echinocytes (Burr cells) before, and after 120 min and 240 min of CPB saline group rats (x40). **(F)** Quantitative measurement of mean echinocyte number/field across 20 fields (*N* = 2). The data shown in **(A–D)** are the mean ± SEM; *N* = 3–4 rats per group. Statistical differences were determined by two-way ANOVA followed by Tukey's test or Student's unpaired *t*-test. ^**^*p* < 0.01 vs. CPB Ghrelin 150 μg/kg (0 min); ^#^*p* < 0.05 vs. CPB Ghrelin 150 μg/kg (protocol 2).

Supplemental Data Figure 2 Raw western blot images.
