# Supplementary figures and images for: Ghrelin Pre-treatment Attenuates Local Oxidative Stress and End Organ Damage During Cardiopulmonary Bypass in Anesthetized Rats
Source: Front Physiol. 2018 Mar 9;9:196. doi: 10.3389/fphys.2018.00196 (PMC5854848; doi:10.3389/fphys.2018.00196)

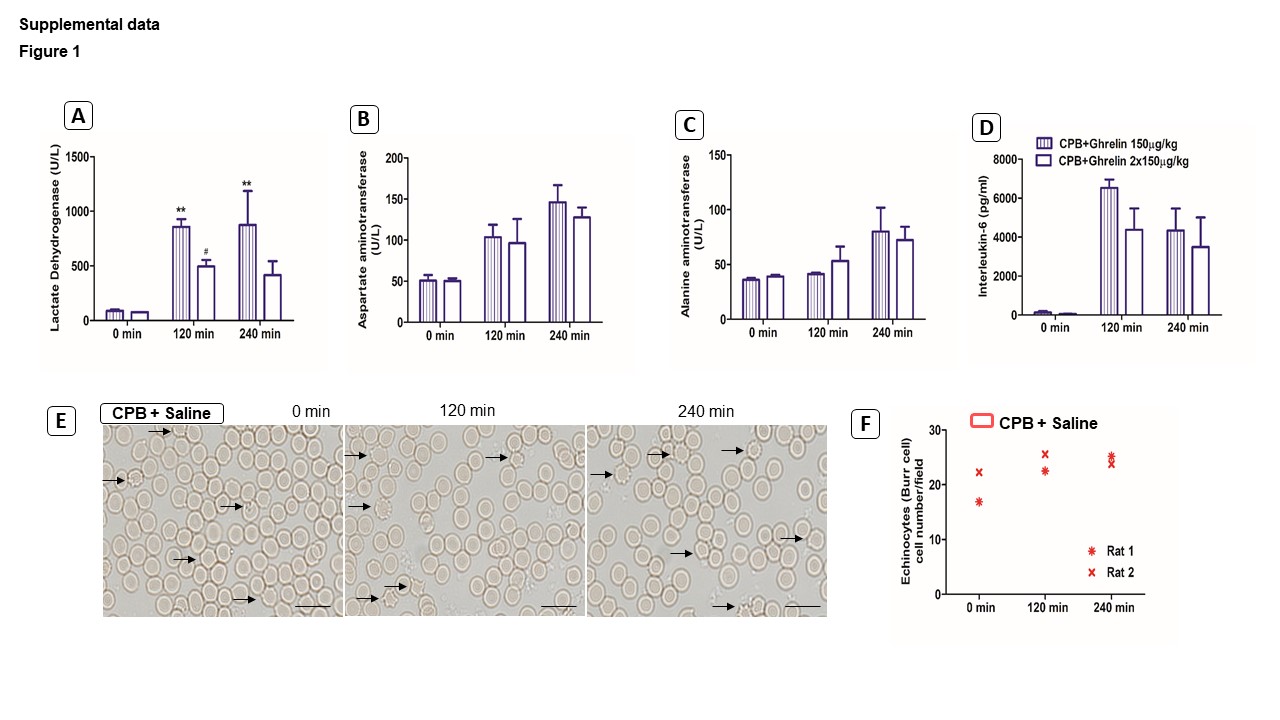

Supplement: Supplemental Data Figure 1 — Effects of single dose ghrelin (protocol 2) versus a double dose of ghrelin (protocol 3) on LDH (A), ALT (B), AST (C), and IL-6 (D) before CPB, and after 120 min of CPB and a further 2 h after recovery from CPB (240 min). (E) Echinocytes (Burr cells) before, and after 120 min and 240 min of CPB saline group rats (x40). (F) Quantitative measurement of mean echinocyte number/field across 20 fields (N = 2). The data shown in (A–D) are the mean ± SEM; N = 3–4 rats per group. Statistical differences were determined by two-way ANOVA followed by Tukey's test or Student's unpaired t-test. **p < 0.01 vs. CPB Ghrelin 150 μg/kg (0 min); #p < 0.05 vs. CPB Ghrelin 150 μg/kg (protocol 2). [file Image1.JPEG]
