# Supplementary material for: Ghrelin Pre-treatment Attenuates Local Oxidative Stress and End Organ Damage During Cardiopulmonary Bypass in Anesthetized Rats
Source: Front Physiol. 2018 Mar 9;9:196. doi: 10.3389/fphys.2018.00196 (PMC5854848; doi:10.3389/fphys.2018.00196)
Supplement: Supplemental Data Figure 2 — Raw western blot images. [file Image2.PDF]

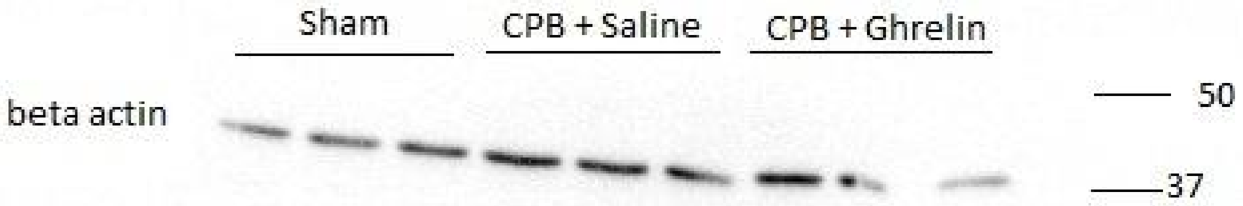
Sham
CPB + Saline
CPB + Ghrelin
beta actin
50
37

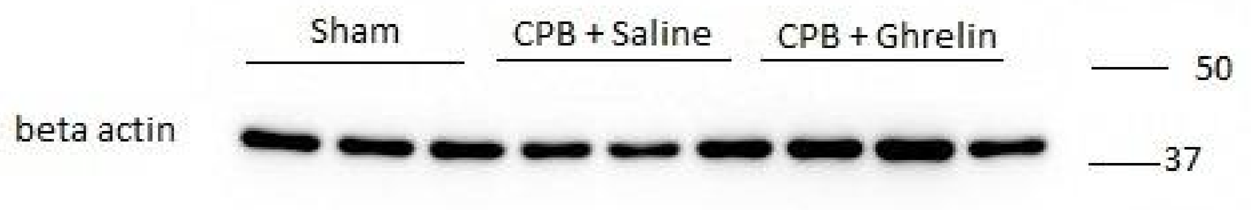
Sham
CPB + Saline
CPB + Ghrelin
50
beta actin
37

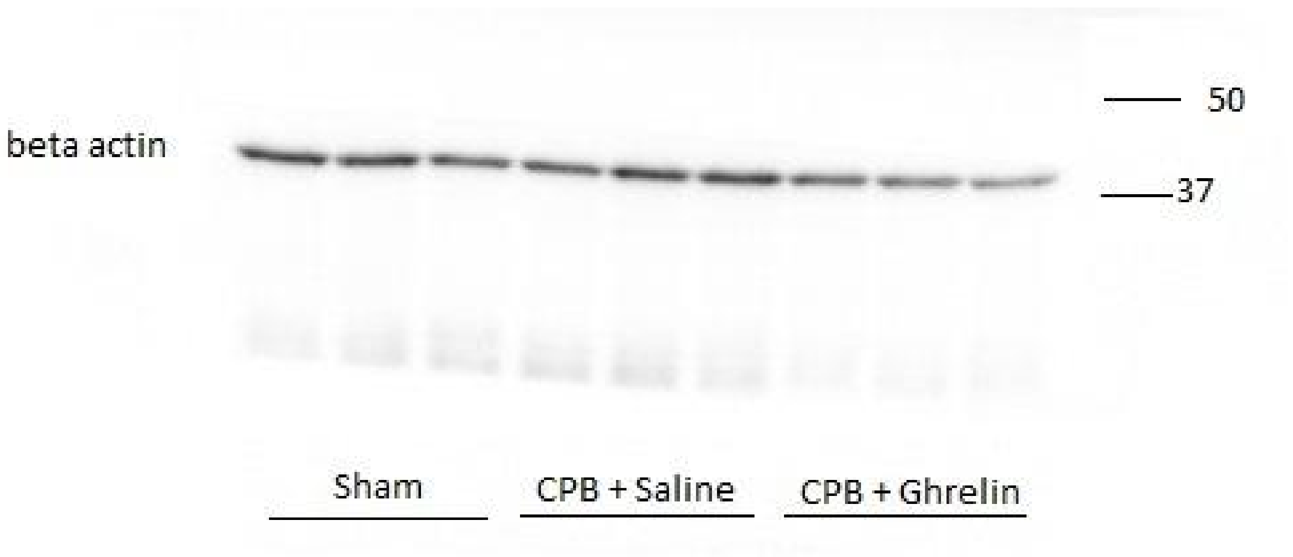

beta actin
50
37
Sham
CPB + Saline
CPB + Ghrelin

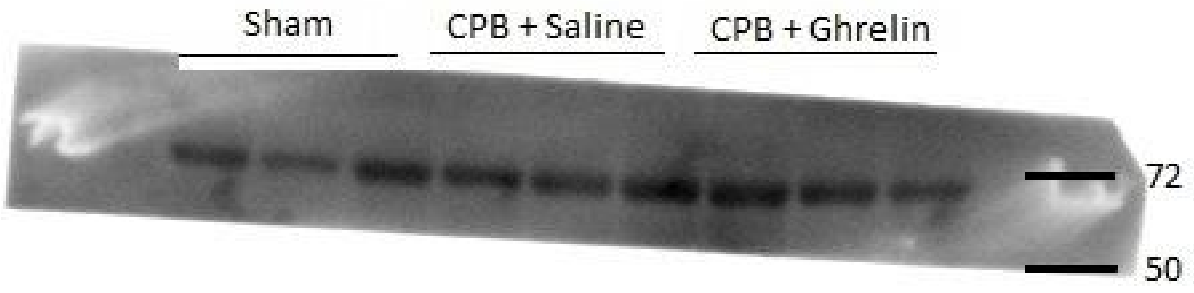
Sham
CPB + Saline
CPB + Ghrelin
72
50

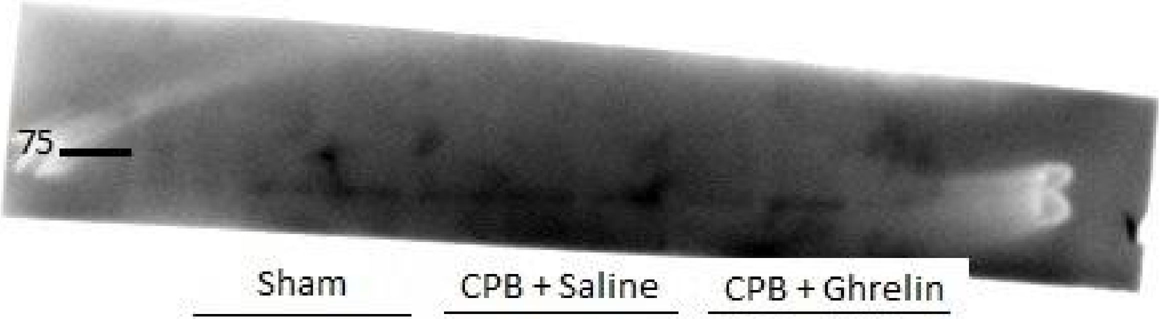
75
Sham
CPB + Saline
CPB + Ghrelin

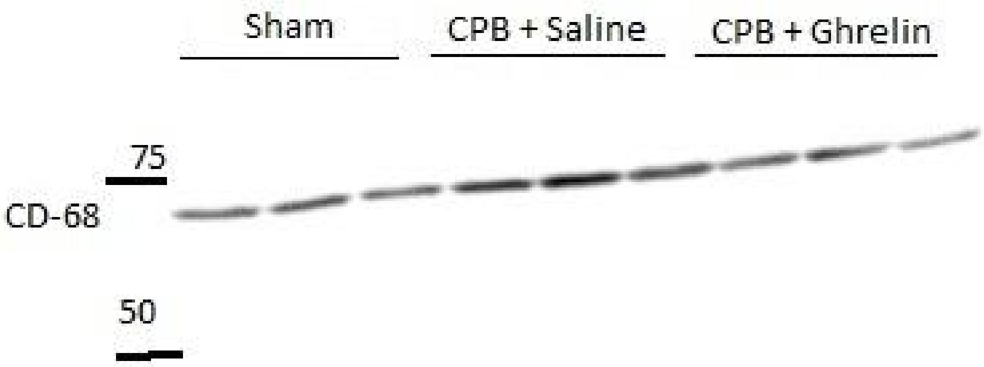
Sham
CPB + Saline
CPB + Ghrelin
75
CD-68
50

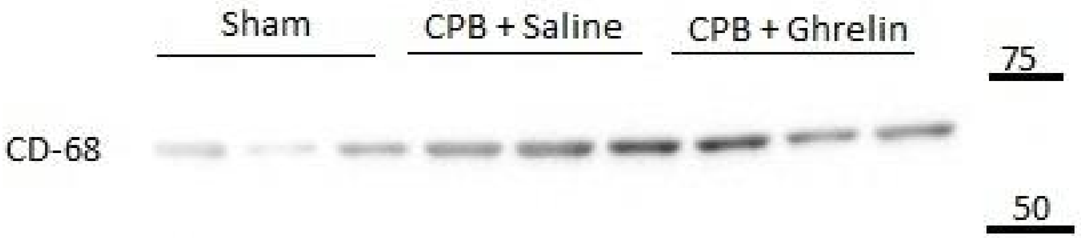
Sham
CPB + Saline
CPB + Ghrelin
75
CD-68
50

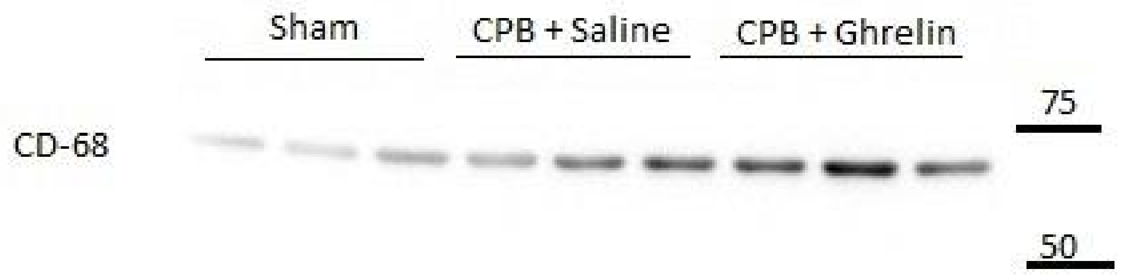
Sham
CPB + Saline
CPB + Ghrelin
CD-68
75
50

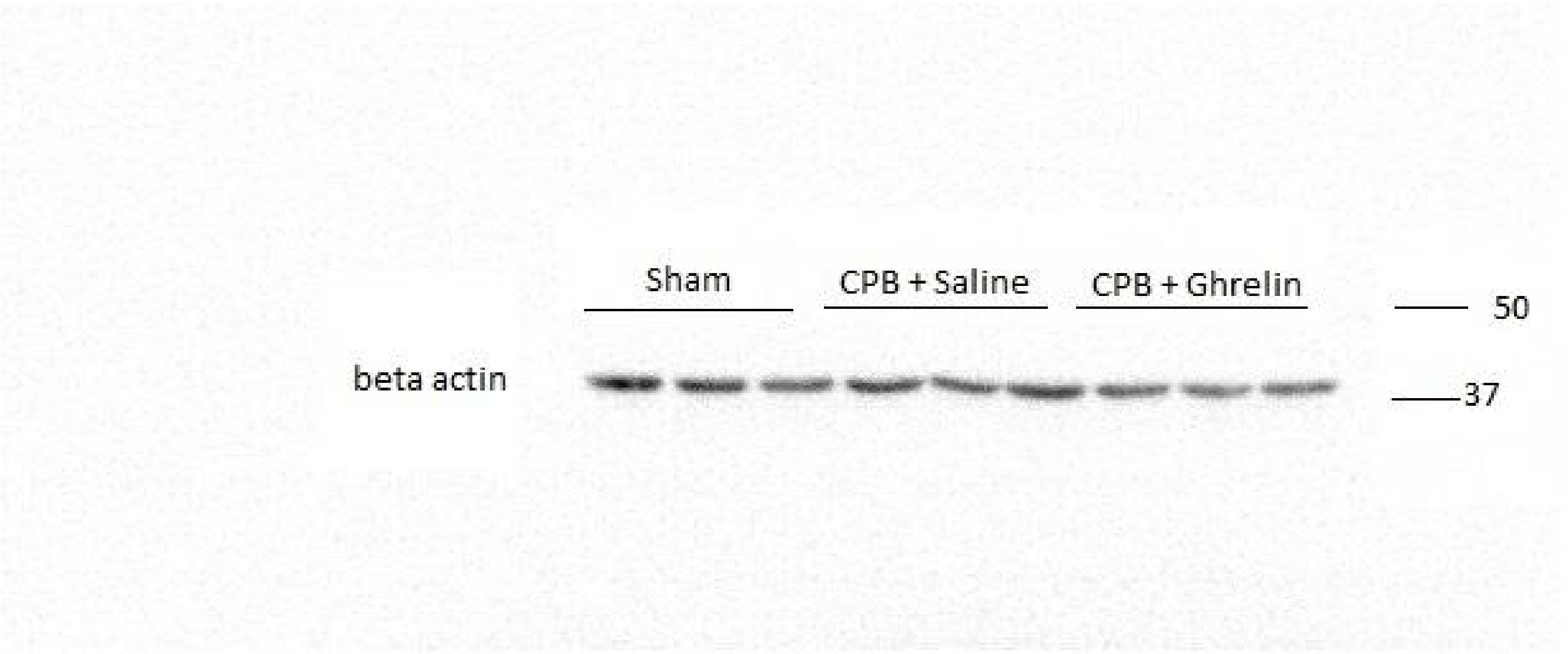

Sham
CPB + Saline
CPB + Ghrelin
50
beta actin
37

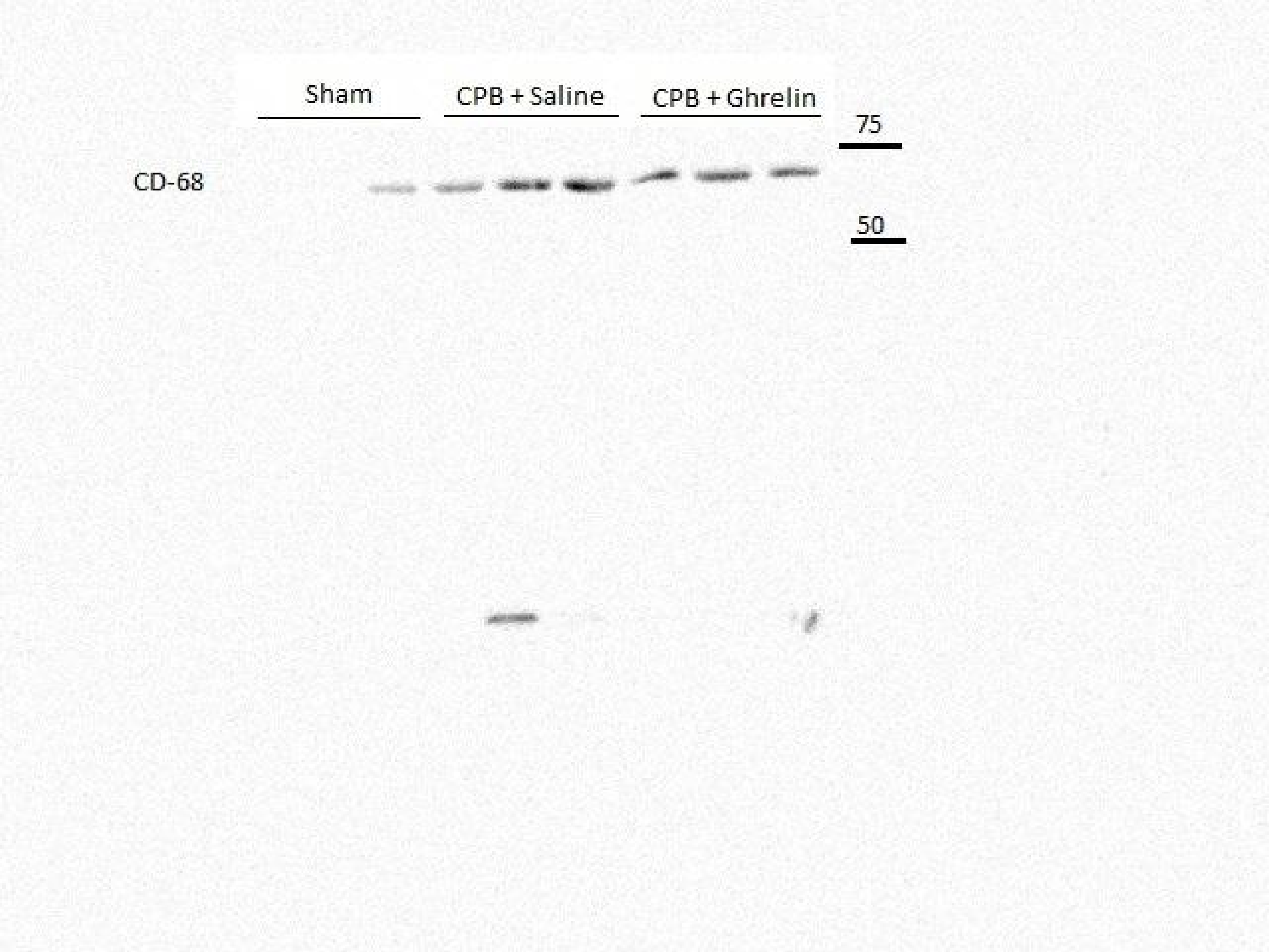

Sham
CPB + Saline
CPB + Ghrelin
75
CD-68
50

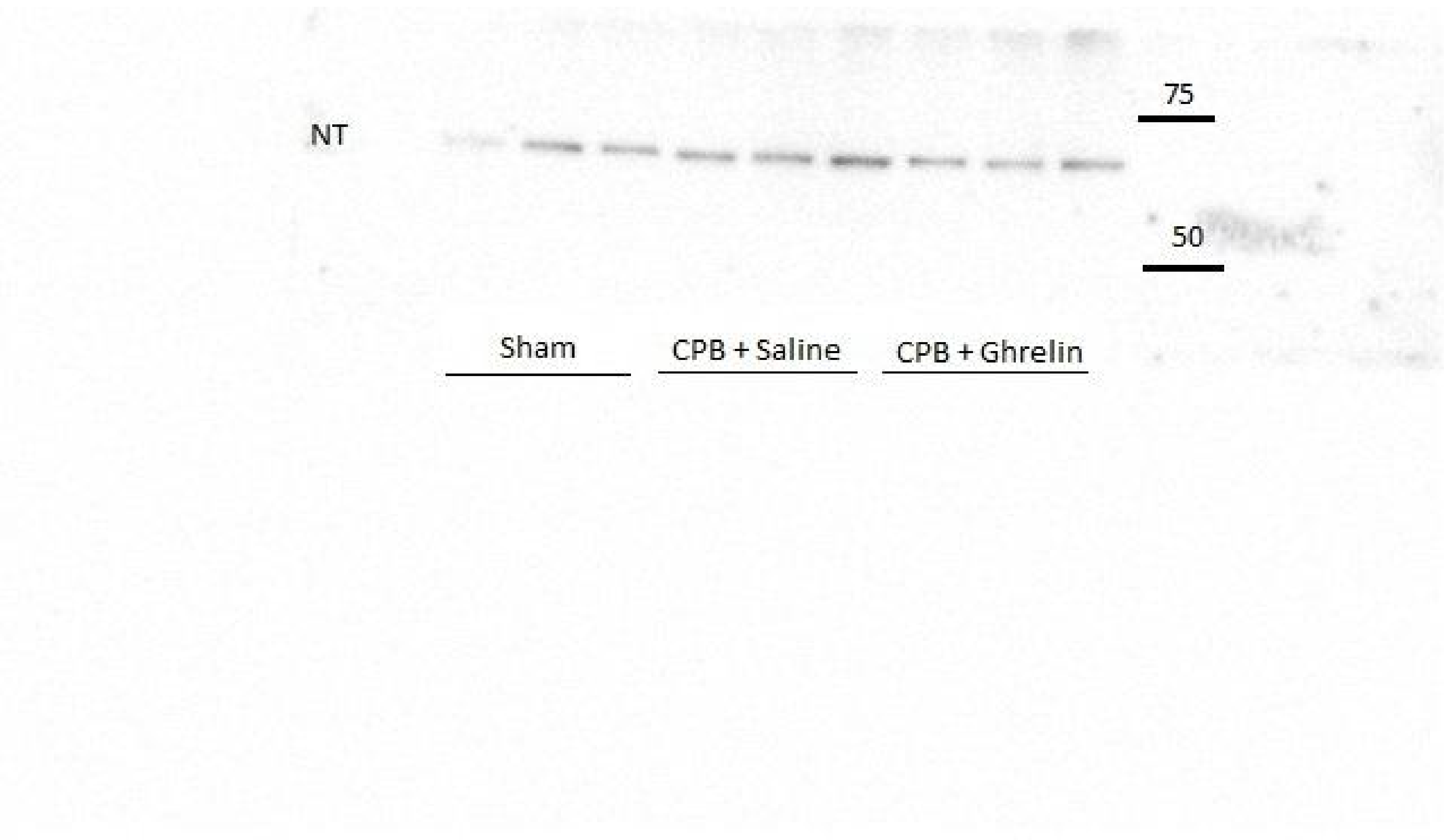
NT
75
50
Sham
CPB + Saline
CPB + Ghrelin

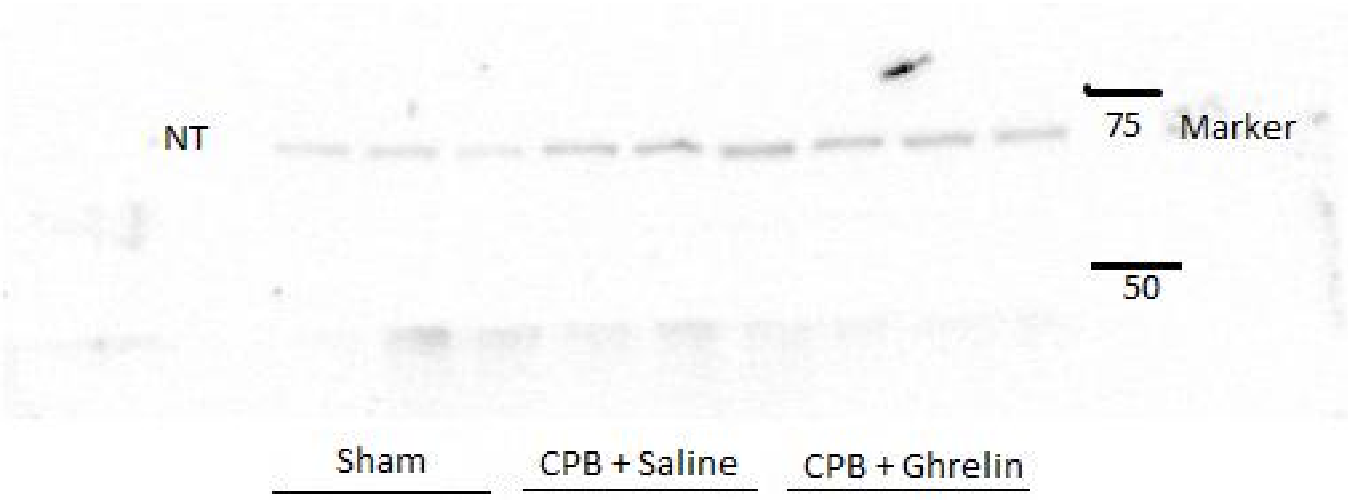
NT
75
Marker
50
Sham
CPB + Saline
CPB + Ghrelin

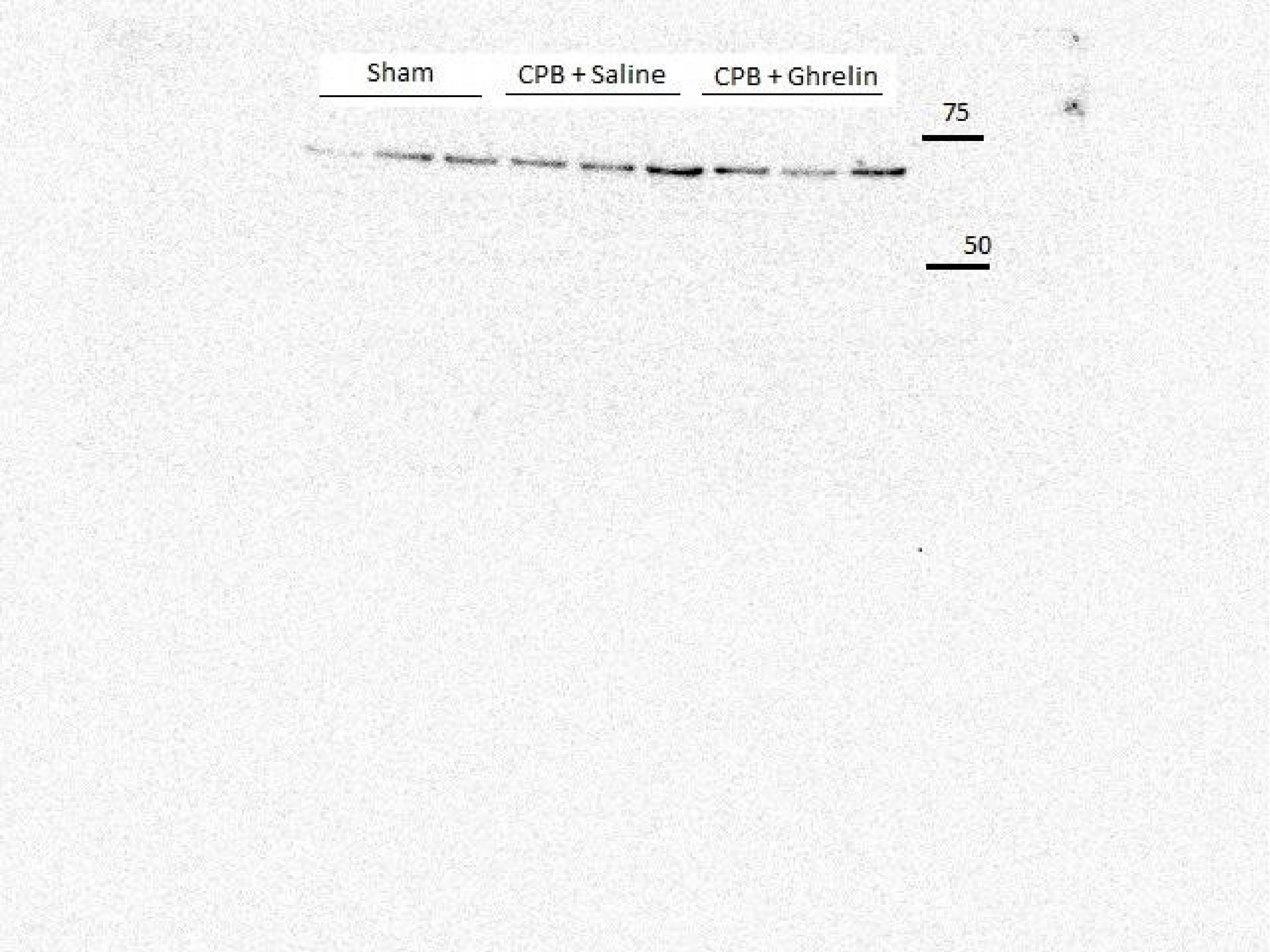
Sham
CPB + Saline
CPB + Ghrelin
75
50

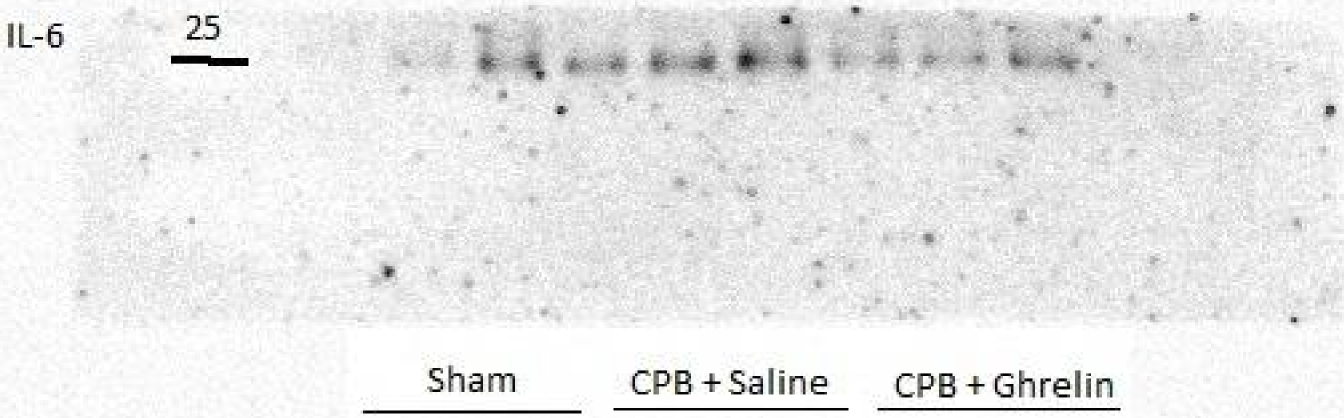
IL-6
25
Sham
CPB + Saline
CPB + Ghrelin

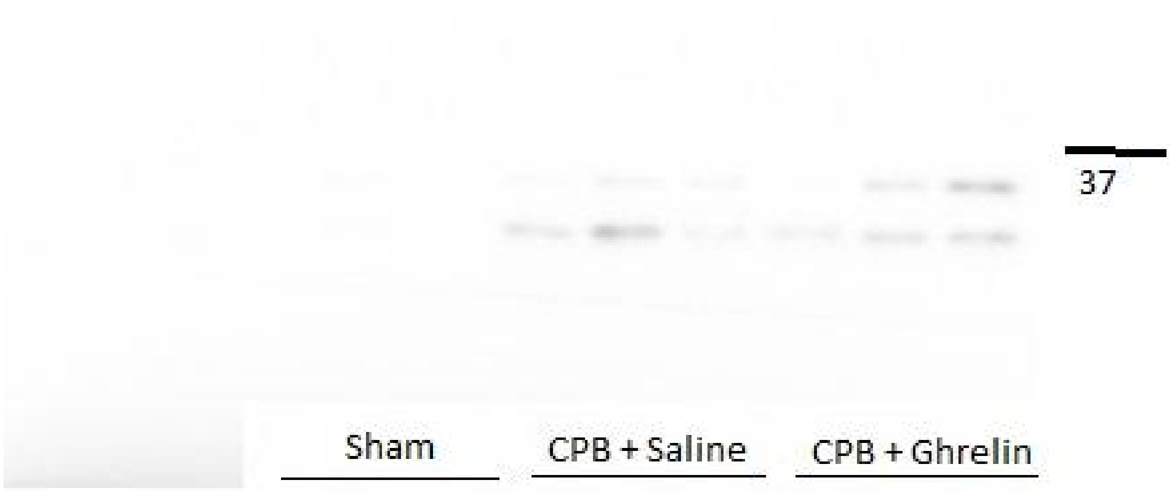
37
Sham
CPB + Saline
CPB + Ghrelin

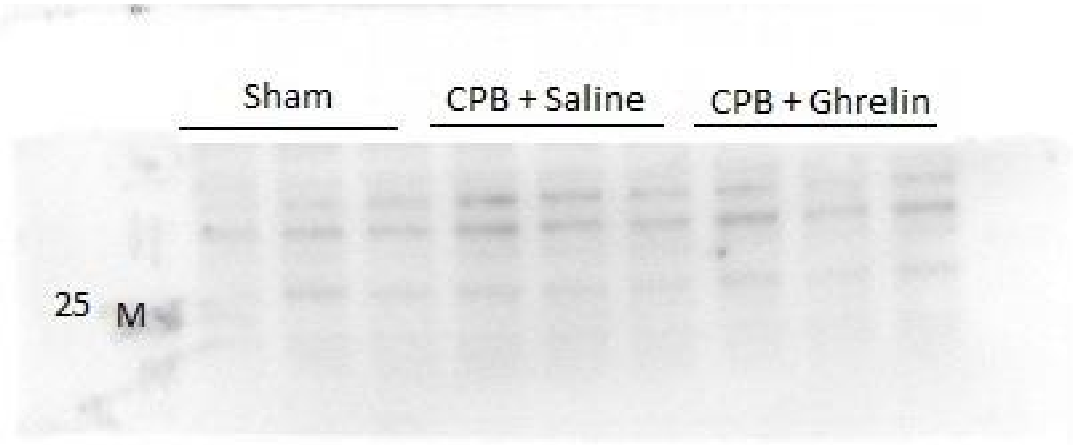
Sham
CPB + Saline
CPB + Ghrelin
25
M

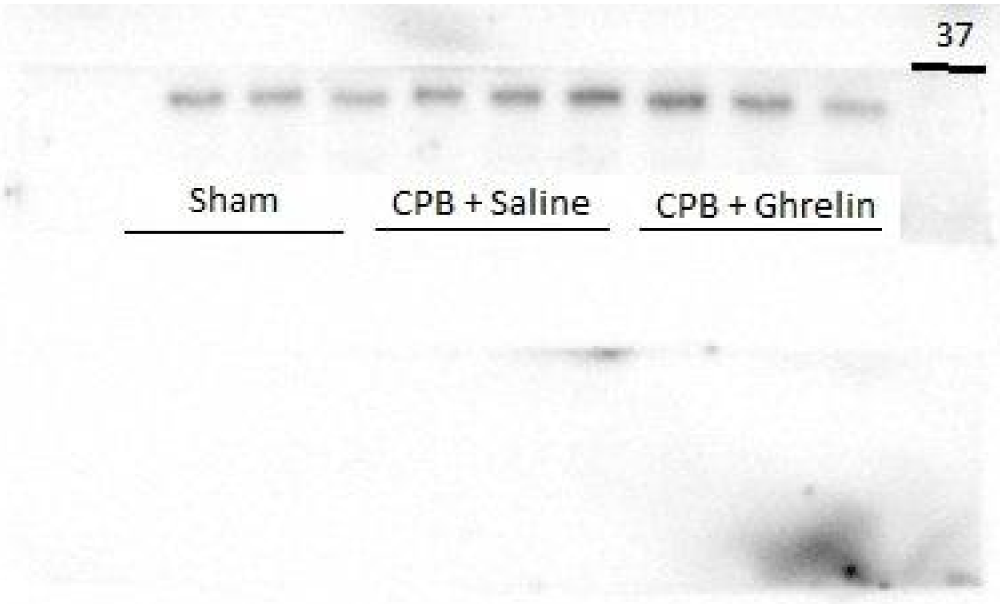
37
Sham
CPB + Saline
CPB + Ghrelin

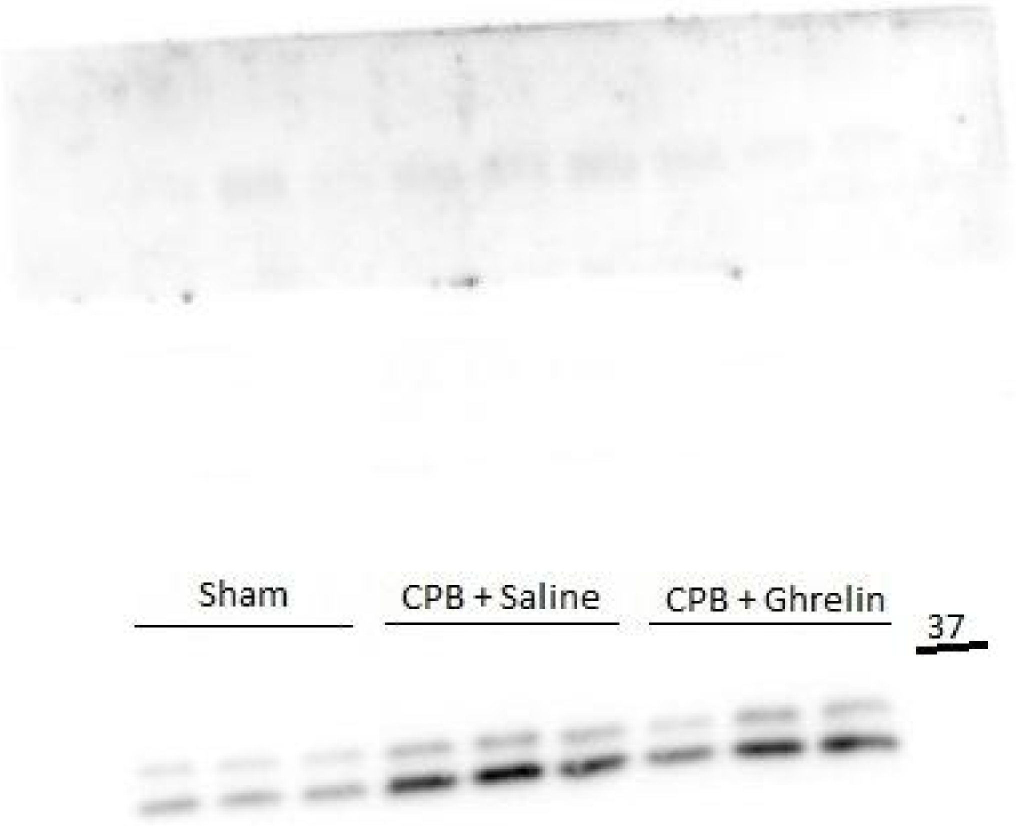
Sham
CPB + Saline
CPB + Ghrelin
37

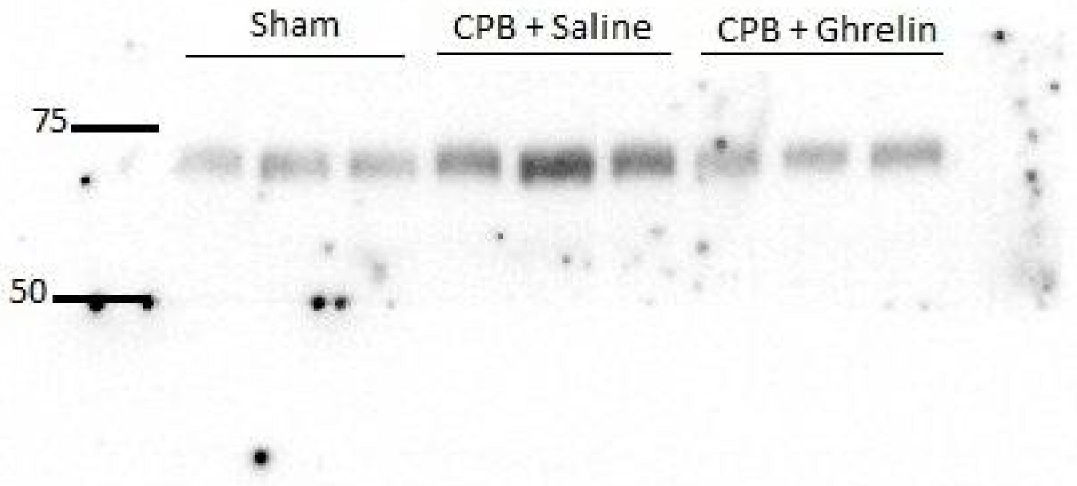
Sham
CPB + Saline
CPB + Ghrelin
75
50

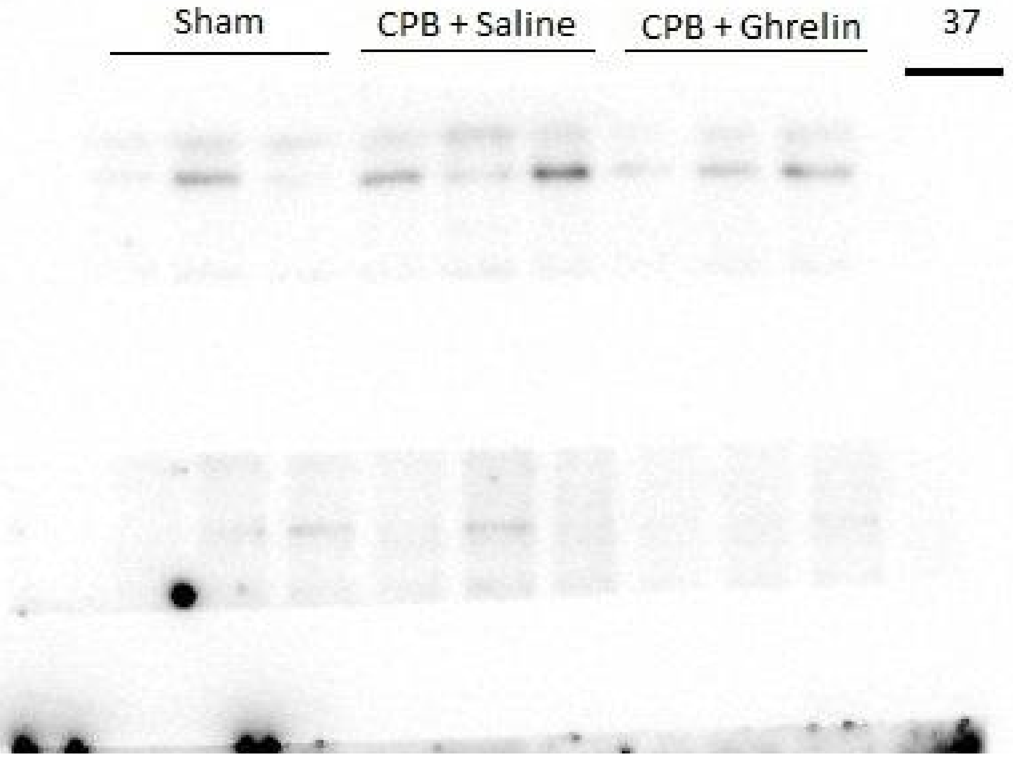
Sham
CPB + Saline
CPB + Ghrelin
37
